# Multi-modal mechanophores based on cinnamate dimers

**DOI:** 10.1038/s41467-017-01412-8

**Published:** 2017-10-27

**Authors:** Huan Zhang, Xun Li, Yangju Lin, Fei Gao, Zhen Tang, Peifeng Su, Wenke Zhang, Yuanze Xu, Wengui Weng, Roman Boulatov

**Affiliations:** 1 Department of Chemistry, College of Chemistry and Engineering, Xiamen University, Xiamen, Fujian, 361005 China; 2 State Key Laboratory of Supramolecular Structure and Materials, College of Chemistry, Jilin University, Changchun, Jilin, 130012 China; 3 Department of Chemistry, University of Liverpool, Donnan Lab, G31, Crown St., Liverpool, L69 7ZD GB UK

## Abstract

Mechanochemistry offers exciting opportunities for molecular-level engineering of stress-responsive properties of polymers. Reactive sites, sometimes called mechanophores, have been reported to increase the material toughness, to make the material mechanochromic or optically healable. Here we show that macrocyclic cinnamate dimers combine these productive stress-responsive modes. The highly thermally stable dimers dissociate on the sub-second timescale when subject to a stretching force of 1–2 nN (depending on isomer). Stretching a polymer of the dimers above this force more than doubles its contour length and increases the strain energy that the chain absorbs before fragmenting by at least 600 kcal per mole of monomer. The dissociation produces a chromophore and dimers are reformed upon irradiation, thus allowing optical healing of mechanically degraded parts of the material. The mechanochemical kinetics, single-chain extensibility, toughness and potentially optical properties of the dissociation products are tunable by synthetic modifications.

## Abstract (web summary)

Mechanochemistry offers exciting opportunities for molecular engineering of stress-responsive properties of polymers. Here the authors show that macrocyclic cinnamate dimers in a polymer chain can undergo dissociation on the sub-second timescale under 1–2 nN stretching to yield a chromophore that then can be optically healed.

## Introduction

A question of intense contemporary interest in polymer chemistry is the development of strategies for molecular-level engineering of material responses to mechanical loads^1–5^. Notable examples include approaches to increase simultaneously the toughness and the elasticity of a polymeric material^6–13^, and to endow the material with the capacity for autonomous indication of internal mechanical damage (e.g., mechanochromism)^14–23^ and self-repair^24–29^. While many demonstrations of individual approaches have been reported, much less effort has been devoted to understanding how they can be integrated to yield materials possessing several of these remarkable properties^30–32^. An example of such a multi-modal mechanochemically-responsive material would simultaneously allow optical identification of overstressed regions; autonomously redistribute local loads in such regions to minimize mechanochemical fragmentation of polymer backbones that can lead to catastrophic material failure; and generate precursors for subsequent reinforcement of these overstressed/weakened regions by photochemical or thermal formation of new load-bearing bonds. While such a material remains to be demonstrated, here we report experimental and computational data suggesting that polymers of macrocyclic dimers of cinnamates are promising candidates to achieve this goal.

The results of our experiments and calculations presented below suggest that single-chain force of 1–2 nN (depending on the isomer) reduces the lifetime of the dimer to the sub-second timescale. This force is sufficiently high for the mechanochemical reaction to occur primarily in overstressed regions of a bulk sample, i.e., regions of high concentration of stress, yet low enough to effectively compete with homolysis of other load-bearing covalent bonds^3,11,33,34^. The large hidden length of the macrocycles released in this dissociation results in high contour-length extensibility (over 200%) and high single-chain toughness (on the order of 600 kcal per mole of monomer), whereas the generated cinnamates make the material optically healable^35^. Simple synthetic modifications of these dimers allow fine tuning of their mechanochemical kinetics; contour-length extensibility and single-chain toughness of their polymers; and potentially absorption/emission spectra of the products of their mechanochemical dissociation. The data presented below extends previous studies of phenylpropenoates for optical healing^29^ and mechanically induced fluorescence^36^, and of the mechanochemistry of cyclobutane cores^8,9,37–39^ to place the macrocyclic dimers reported here among the more promising candidates to yield multi-modal stress-responsive polymers.

## Results

### DFT calculations of reaction mechanisms and energies

**Fig. 1:**
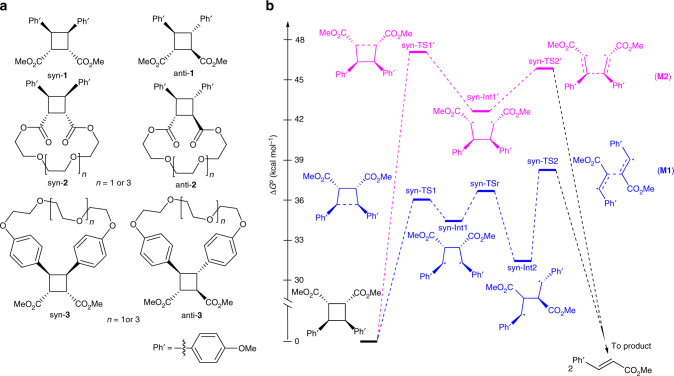
Cinnamate dimers and the strain-free dissociation mechanism. **a** The chemical structures of the studied cyclobutane derivatives. **b** Mechanisms of dissociation of syn-**1** calculated at the uMPW1K/6-31 + G (d) level of the DFT in vacuum. The barrier for rotation around the Ph’C–CPh′ bond of syn-Int’ is comparable to that for syn-Int (3.5 kcal mol^−1^) but the corresponding rotated syn-TS2’_r_ conformer is 1.8 kcal mol^−1^ less stable than syn-TS2’ and bond rotation in syn-Int’ is only important under force (see Supplementary Figs. 1–3 for dissociation mechanisms of anti-**1** with and without force)

To identify cinnamate dimers best suited for stress-responsive applications, we calculated the mechanisms and activation free energies of dissociation of syn and anti isomers of head-to-head dimers of methyl *E*-3-(4-methoxyphenyl)acrylate (a derivative of cinnamic, or 3-phenylacrylic acid) and its macrocyclic derivatives (Fig. 1a) as a function of applied force at the uMPW1K/6-31 + G(d) level of DFT in vacuum using a well-established methodology^38,40–49^. We chose the uMPW1K/6-31 + G(d) model chemistry because it reproduced with useful accuracy the electronic activation energies of dissociation of a related derivative, cyclobutane-1,2-dicarboxylic acid, calculated at the uCCSD/jun-cc-pVTZ//uCCSD/6-31 G* level of theory (Supplementary Table 1). Thermal dissociation of cyclobutanes to olefins is a formally symmetry-forbidden [2 + 2] cycloreversion and our calculations indicate that it proceeds by a radical stepwise mechanism (Fig. 1b for syn-**1** and Supplementary Fig. 1 for anti-**1**), as confirmed experimentally for a related derivative under force^8^. In the absence of force, the minimum-energy pathway (**M1**, blue Fig. 1b) starts with homolysis of the phenyl-substituted C–C bond, followed by the rate-determining dissociation of the second scissile bond. Dissociation of syn-**1** has a total activation free energy, Δ*G*_t_^‡^, of 38.4 or 6.4 kcal mol^−1^ lower than that of anti-**1** (44.8 kcal mol^−1^). Δ*G*_t_^‡^ of an alternative dissociation mechanism (**M2**, magenta, Fig. 1b) in which the bond bearing the ester groups dissociates first is 8.0 kcal mol^−1^ (syn) and 8.6 kcal mol^−1^ (anti) higher than **M1**.

**Fig. 2:**
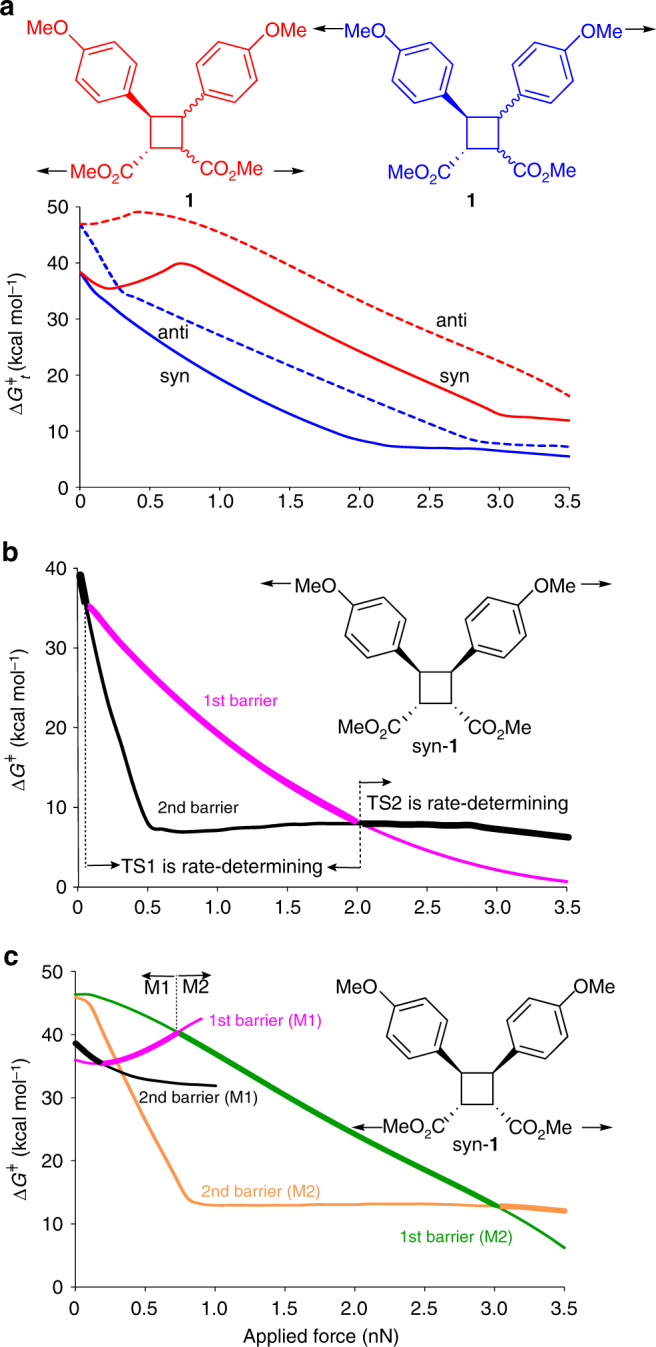
The calculated effect of force on the barriers of dimer dissociation. **a** Total activation free energies of dissociation, Δ*G*_t_^‡^, of syn-1 and anti-1 for two pulling axes defined by the arrows. **b** Free energies of the 1st (magenta) and 2nd (black) barriers of syn-**1** dissociation pulled on the C_Me_ atoms of methoxyphenyl groups. **c** The free energy barriers of **M1** (magenta and black) and **M2** (green and orange) dissociation mechanisms of syn-**1** pulled on the C_Me_ atoms of carboxymethyl groups. In **b**, **c**, the segments of force-dependent energies corresponding to the rate-determining barriers are shown in bold. All energies are at uMPW1K/6-31 + G (d) in vacuum. The force-dependent barriers for dissociation of anti-**1** are illustrated in Supplementary Fig. 2. See also Supplementary Fig. 3 for complete energy profiles along the reaction paths at select forces

Our calculations indicate that pulling the dimers at the methyl C atoms of either methoxyphenyl or carboxymethyl groups (Fig. 2a) accelerates their dissociation, albeit with considerably different mechanochemical kinetics. The complex dependence of Δ*G*_t_^‡^ on force (Fig. 2a) results from the much larger stabilization by force of the intermediate and the second transition states compared to the first transition state. This differential stabilization causes the rate-determining step to change as the force increases (Fig. 2b, c), with concomitant decreases in the slopes of the Δ*G*_t_^‡^(*f*) correlations^48^. For example, in dissociation of syn-**1** pulled at the C_Me_ atoms of the methoxyphenyl groups such changes occur at 0.12 nN, 0.52 nN and 2.1 nN (black vs. magenta curves, Fig. 2b and Supplementary Fig. 3a), when, respectively, syn-TS2_r_ becomes more stable than syn-TS1, syn-Int_r_ becomes more stable than the reactant and the energy difference syn-TS1 and the reactant (1st dissociation barrier) decreases below that of syn-TS2_r_ and syn-Int_r_ (2nd dissociation barrier). Below 0.12 nN the rate is limited by the energy difference between syn-TS2_r_ and the reactant; between 0.12 nN and 2.1 nN the rate-determining difference is between of syn-TS1 (which is now less stable than syn-TS2_r_) and the reactant; above 2.1 nN, the energy difference between syn-Int_r_ and syn-TS2_r_ limits the dissociation kinetics. Correspondingly, the slope of Δ*G*_t_^‡^(*f*) (solid blue curve, Fig. 2a) first decreases at 0.12 nN because syn-TS1 is shorter than syn-TS2_r_ along the pulling axis^34,47,50,51^ and then reduces to ~ 0 at 2.1 nN (i.e., Δ*G*_t_^‡^ becomes force-independent) because the two relevant states (syn-TS2_r_ and syn-Int_r_) have nearly identical _Me_C^…^C_Me_ separations at the same force. Because the 2nd is force-independent but the 1st barrier decreases monotonically with force, the change in the rate-determining barrier occurs. In other words, force >2.1 nN barely affects the rate at which the dimers dissociate. The same sequence of changes of the rate-determining barriers accounts for Δ*G*_t_^‡^(*f*) of dissociation of anti-**1** pulled at the C_Me_ atoms of the methoxyphenyl groups (Supplementary Figs. 2, 3). Energy profiles along the dissociation paths at different forces shown in Supplementary Fig. 3 illustrate these changes.

The even more complex, non-monotonic force-dependence of Δ*G*_t_^‡^ for dissociation of **1** pulled on the C_Me_ atoms of the carboxymethyl groups (red lines, Fig. 2a) reflects the competition between the two dissociation mechanisms (**M1** and **M2**, Fig. 1b) and the two transition states of each mechanism (Supplementary Fig. 3c, d). Whereas pulling syn-**1** on the methoxyphenyl groups stabilizes all states of the minimum-energy **M1** dissociation mechanism (Fig. 2b), force applied to the carboxymethyl groups destabilizes syn-TS1 of **M1** (magenta line, Fig. 2c). Despite this destabilization, the energy of **M1** remains below that of **M2** up to 0.83 nN and dissociation of syn-**1** pulled at the carboxymethyl groups with <0.83 nN follows the **M1** mechanism. As a result, force between 0.25 and 0.83 nN pulling on the carboxymethyl groups inhibits dissociation of syn-**1** (see also Supplementary Figs. 2, 3). At 0.83 nN, the dissociation rate reaches the minimum when the rate-determining transition states of **M1** and **M2** mechanisms are isoenergetic (green vs. magenta lines, Fig. 2c and Supplementary Fig. 3c, d). Above 0.83 nN, dissociation follows the **M2** mechanism and is accelerated by force up to 3 nN. Above 3 nN, the rate-determining transition state changes from syn-TS1’ to syn-TS2’_r_ (orange line, Fig. 2c) and the dissociation rate becomes approximately force-independent.

Pulling on the carboxymethyl groups is considerably less efficient in accelerating the dissociation: e.g., at 2 nN and 300 K, anti-**1** dissociates >10^12^-fold faster when pulling on phenoxy-bound instead of ester-bound methyl groups; for syn-**1**, the ratio is >10^11^. Alternatively, an extra 1.5 nN is required to reduce the half-life of either isomer of **1** to 1 s if one pulls on the carboxymethyl groups rather than on the methoxyphenyl moieties. This difference explains the recently reported difficulty of inducing mechanochemical dissociation of cyclobutane derivatives pulled at the carboxymethyl groups^37^.

The computational data summarized above demonstrate that dissociation of suitably designed cinnamate dimers can be sufficiently accelerated by stretching force to compete effectively with non-selective load-induced dissociation of polymer backbones (Supplementary Fig. 4)^52^. To ensure that dimer dissociation increases rather than reduces the number of load-bearing bonds in the loaded material, we designed macrocyclic derivatives **2** and **3** (Fig. 1a), in which a polyether linker bridges a pair of the phenoxy or carboxy groups. The linker represents these dimers’ hidden length, whose release in the mechanochemical reaction increases the contour length of the stretched polymer and hence the mechanical strain energy that the chain can sustain without failure^6,11^. In this respect, the macrocycle design mimics the structural basis of the remarkable micromechanics of the structural protein titin^53^. We chose polyether linkers to exploit alkali-ion templating in dimer synthesis (see the next section). We calculated the effect of linkers of two lengths (*n* = 1 and 3, Fig. 1a) on the mechanochemical reaction mechanisms and dissociation kinetics based on our preliminary experimental findings that the strap length affects the yield of photodimerization and the solubility of the resulting polymers.

The primary effect of bridging either the carboxylate or phenoxy groups of the cinnamate dimers is to destabilize the rotated conformers of the 2nd transition state (TS2_r_ in Fig. 1b, and Supplementary Figs. 1 and 3). In series **2** macrocycles (carboxylates are bridged), this destabilization slightly inhibits dissociation at forces >1 nN for the shortest linker (*n* = 1, Supplementary Fig. 5). In series **3** macrocycles (phenoxy groups are bridged), this destabilization results in dimers pulled with >0.5 nN dissociating to *E,Z* dienes instead of the *E,E* dienes generated by dissociation of dimers **1** and **2** or of dimers **3** at <0.5 nN (Supplementary Fig. 6). The product stereochemistry is determined by the relative energies of the conformers of the product-determining (2nd) transition state generated by rotation around either the 2nd scissile or one of the non-scissile bonds of the cyclobutane core (Supplementary Fig. 7). This rotation increases the length of the 2nd transition state along the pulling axis and hence is favored by applied force. In series **3**, the steric requirements of the linker negate the force stabilization from rotation around the 2nd scissile bond but not from rotation around the non-scissile bond, making the latter conformers the preferred reaction path leading to *E,Z* dienes. The same effect is responsible for mechanochemical dissociation of dicarboxycyclobutene derivatives fused to cyclohexane or cyclooctane, which prevents rotation around the 2nd scissile bond in the intermediate, yielding *E,Z* dienes at high force^9,38^. In addition, linking the phenoxy groups (series **3**, Fig. 1a) significantly strains the anti isomers of the resulting macrocycles, so that anti-**3** dimers may be synthetically inaccessible. This conclusion is supported by a recently reported failure to obtain a macrocyclic dimer similar to anti-**3** with *n* = 4^37^.

**Fig. 3:**
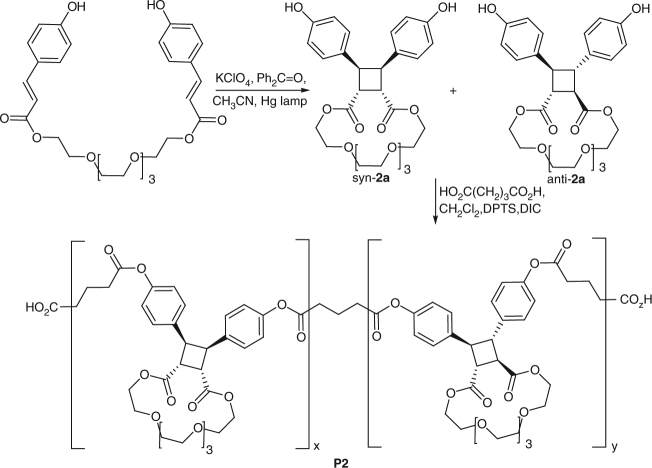
Synthesis of macrocyclic dimers **2a** and their copolymer **P2**. DPTS: dimethylaminopyridinium toluenesulfonate, DIC: *N*,*N*-diisopropylcarbodiimide

The computational results summarized above suggest that linking the carboxy groups of cinnamate dimers (series **2**) is a significantly more promising approach to building up hidden length than linking the phenoxy groups (series **3**). Compared to series **3**, macrocycles **2** dissociate with more favorable mechanochemical kinetics into *E,E* dienes so that the same stereoisomers of the dimers are photochemically regenerated (whereas mechanochemical dissociation and optical regeneration of series **3** macrocycles scrambles the stereoisomers) and linking the carboxylate groups does not strain the resulting dimers, unlike anti-**3** whose strain probably makes them synthetically inaccessible. Of the two linkers studied computationally, we chose the longer one (*n* = 3, Fig. 1a) to study experimentally because it provided the right balance between dimerization yield (which decreases with the linker length) and the favorable mechanochemical dissociation kinetics and single-chain toughness (which favors longer linkers). Below we refer to these dimers as syn-**2a** and anti-**2a** (Fig. 3).

### Synthesis and thermal stability

We synthesized a mixture of syn-**2a** and anti-**2a** (Fig. 3) in 1:5.2 molar ratio and 47% yield by K^+^-templated intramolecular [2 + 2] photocyclization^54,55^ of 3,6,9,12-tetraoxatetradecane-1,14-diyl (2E,2′E)-bis(3-phenylacrylate) (see Supplementary Fig. 8 for further details). The resulting mixture was reacted with excess glutaric acid to yield a random copolymer of syn-**2a** and anti-**2a**, **P2**, whose chemical composition, including the syn/anti ratio of the monomers, was established by^1^H NMR spectroscopy (*M*_n_ = 54 kDa, PDI = 1.3, Supplementary Fig. 9). We chose to co-polymerize the dimers because a copolymer allowed us to acquire twice as many force/extension curves for each dimer as individual polymers would have.

The high thermal stability of the cinnamate dimers preclude accurate determination of the activation free energies of dissociation but qualitative observations are consistent with the results of our quantum-chemical calculations suggesting negligible dissociative lability (*t*_½_ > 1 h) up to 300 °C (Supplementary Fig. 10). First, NMR spectra of a solution of **P2** didn’t change after 27 h at 150 °C. Second, differential scanning calorimetry measurements revealed no signatures of chemical reaction up to 250 °C. Third, thermogravimetric analysis of **P2** revealed only pyrolysis at >400 °C under anaerobic conditions. These finding are consistent with the previous attempts to estimate the kinetics of thermal dissociation of the cyclobutane cores^8,9^ and the results of previous quantum-chemical calculations^38^.

### Experimental single-chain mechanochemistry

**Fig. 4:**
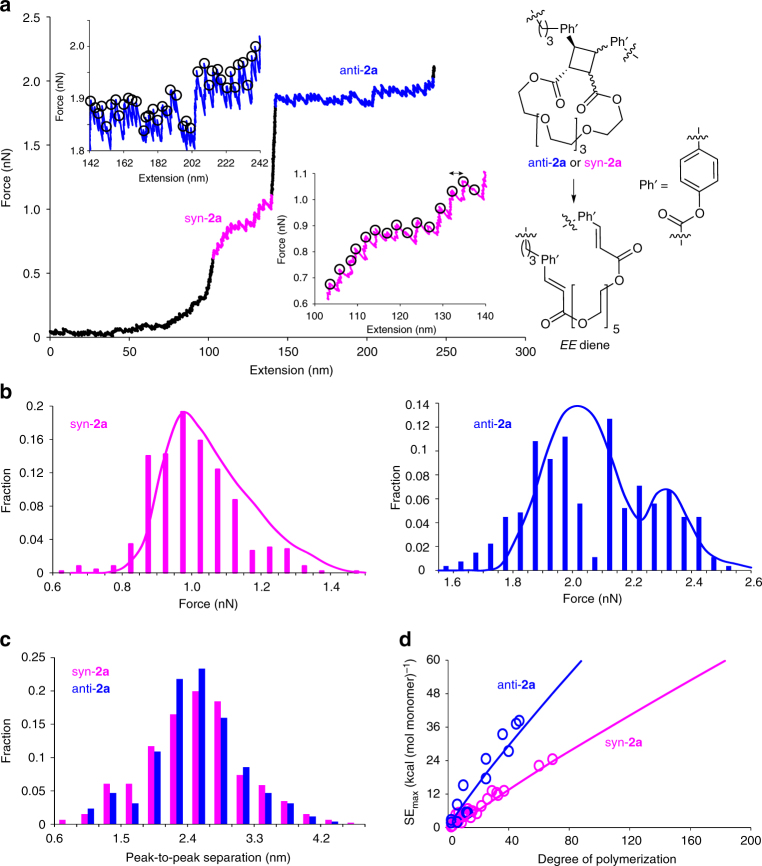
Summary of single-chain mechanochemistry of macrocyclic cinnamate dimers. **a** A representative force/extension curve of copolymer **P2**. The two plateaus, resulting from dissociations of syn-**2a** and anti-**2a** to *E,E* diene are highlighted in magenta and blue, respectively. Magnified plateau regions in the insets illustrate the saw-tooth pattern corresponding to dissociation of individual dimers, identified by black circles. **b** The experimental (bars) and computed (solid lines) distribution of dissociation forces at the 1st (magenta) and the 2nd (blue) plateaus. **c** The observed distribution of peak-to-peak separations, corresponding to an increase in the polymer contour length upon dissociation of individual dimers. **d** Calculated (solid lines) and experimentally derived (circles) maximum strain energies that a single macromolecule of syn-**2a** (magenta) or anti-**2a** (blue) can sustain before failure

We quantified single-chain mechanochemistry of copolymer **P2** by constant-velocity single-molecule force spectroscopy (SMFS)^56–59^. The experiments were performed in dimethylformamide with the polymers anchored to a silicon slide at one terminus and to an AFM tip at the other by amide bonds. The retraction rate of the AFM was 1 μm s^−1^. A representative force-extension curve is shown in Fig. 4a (additional curves are shown in Supplementary Fig. 11). The curve has two plateaus (highlighted in magenta and blue), where the polymer contour length increases due to mechanochemical dissociation of macrocyclic cinnamate dimers. The well-resolved saw-tooth pattern of each plateau (insets) reveals dissociation of individual monomers. Similar plateaus are seen in force/extension curves of other multi-mechanophore polymers^11,33,38^, although in the past reactions of individual sites could not be resolved. Figure 4b shows the distribution of forces at which the 461 transitions at the 1st plateau and the 257 transitions at the 2nd plateau occurred in the 30 measured force/extension curves.

We ascribed the 1st and the 2nd plateaus to mechanochemical dissociations of the syn and anti dimers, respectively. This interpretation is based on the good agreement (Fig. 4b) of the experimental distribution of transition forces with that obtained from 1.5 × 10^9^ unique force/extension curves calculated for the same set of 30 macrochains studied experimentally using the force-dependent free energies of dissociation presented above (Fig. 2 and Supplementary Figs. 4 and 5), as detailed in the Quantum-mechanical calculations section of the Supplementary Information. These calculations predicted the most likely dissociation force of the syn dimer in our experiments of 0.95 nN vs. 1.0 nN when the observed forces are analyzed as a discrete distribution with 50-pN-wide bins. The same numbers are 2.0 nN and 2.1 nN for the anti dimer. The root-mean-square deviation between the predicted and observed probability distributions is 0.03 and 0.04 for syn and anti dimers, respectively.

Our simulations of force/extension curves using quantum-chemically calculated activation free energies reveal that single-chain mechanochemical kinetics depends strongly on the chain length and the number of equivalent reactive sites in the chain, in addition to the usually considered stretching rate (Supplementary Fig. 12)^33,34,46,60^. For example, our calculations indicate 85% probability of at least 35 syn dimers of the longest chain fragment we stretched (69 syn and 653 anti dimers) dissociating below 1 nN. Experimentally, the 35th syn dimer dissociated at 0.87 nN. In contrast, we computed only 2% probability of at least 1 syn dimer dissociating below 1 nN in a shorter fragment of 15 syn and 383 anti dimers. In agreement, the lowest transition force observed was 1.03 nN.

This dependence of the reaction probability on the number of equivalent reaction sites also explains the approximately lognormal distribution of the dissociation force of syn-**2a** (Fig. 4b, magenta). Our calculations indicate that in a stretched macrochain containing multiple (>15) equivalent reactive sites, the first few sites react with approximately equal probability at the same applied force, but as the number of unreacted sites decreases, larger and larger force is required to maintain the same dissociation probability. The result is a distribution with a long high-force tail (e.g., black and green lines in Supplementary Fig. 12). The distribution of dissociation forces of the anti dimers is bimodal, with the low-force component comprised of chains that detached after only ~ 5% of their anti dimers dissociated. The high-force component results from the three shortest chains in which >50% of the anti dimers dissociated before detachment (blue, red and green curves in Supplementary Fig. 12b). When we modeled force/extension curves without allowing chain detachment to complete with mechanochemical dissociation of anti-**2a**, the distribution of dissociation forces became approximately lognormal (Supplementary Fig. 13).

The measured peak-to-peak separations of individual mechanochemical transitions (double-headed arrow, Fig. 4a inset), which correspond to the increase in the chain contour length upon dissociation of individual dimers, were distributed approximately normally around 2.5 nm with the variance of 0.7 and 0.5 nm for syn-**2a** and anti-**2a**, respectively (Fig. 4c). The corresponding elongations calculated at the uMPW1K/6-31 + G(d) level are 2.46 and 2.49 nm at 1 nN and 2 nN (median transition force in experiment) for dissociation of syn-**2a** and anti-**2a** to the *E,E* diene, respectively. These increments correspond to more than doubling of the contour lengths of poly(syn-**2a**) or poly(anti-**2a**) at 1 nN and 2 nN, respectively.

While a macrochain containing the dimers can be stretched reversibly up to the force below the dimer dissociation force (e.g., red and green lines, Supplementary Fig. 14a), elongation of the contour length of the stretched chain associated with the plateau is irreversible (blue vs. magenta lines in Fig. 4a). Reversing the direction of the translation of the AFM tip when the plateau is traversed does not retrace the original force/extension curve but produces a new curve corresponding to a longer chain. Similarly, force/extension curves of a polymer of cinnamates, instead of cinnamate dimers, do not manifest any plateaus (Supplementary Fig. 14b). These observations further support our conclusion that the plateaus result from a release of the hidden length of the dimers due to their dissociation.

An increase in the contour length of the chain upon dissociation of individual dimers momentarily reduces the load the chain experiences (as illustrated by the drops in the force recorded in SMFS experiments after each dissociation, Fig. 4a insets) and increases the amount of strain energy that the chain can absorb without fragmenting, SE_max_. The latter places the upper limit on the material toughness^61^, which is the strain energy per unit volume that the material can absorb before fracturing. Experimentally, single-chain SE_max_ is the area under the force/extension curve up to chain fragmentation (or a failure of a bond between the chain and one of the surfaces, as these two events cannot be experimentally distinguished in our SMFS experiments). Figure 4d compares the calculated most-probable SE_max_ of analogs of **P2** containing only syn-**2a** or only anti-**2a** as a function of the chain size with equivalent SE_max_ extracted from the experimental force/extension curves of copolymer **P2**. The good agreement between the experiments and the calculations further supports our assignments of the reactions responsible for the two plateaus (Fig. 4) and the calculated mechanochemical profiles (Fig. 2 and Supplementary Figs. 5 and 6), and allows us to draw some general conclusions about the capacity of polymers of macrocyclic cinnamate dimers to resist fragmentation under tensile load. First, our calculations indicate that per monomer single-chain SE_max_ decreases approximately proportionally to a log of the number of monomers (degree of polymerization, Supplementary Fig. 15), because mechanochemical reactions occur at a lower force in longer chains comprised of more equivalent reactive sites. Second, SE_max_ is calculated to increase with constant stretching rate, because the faster the stretching rate, the higher force can be reached before dimers dissociate and the chain breaks. Third, relative to a hypothetical polymer of the same contour length and compliance as a polymer of **2a** but lacking mechanochemically labile reactive sites, syn-**2a** and anti-**2a** mechanophores increase single-chain SE_max_ by >5-fold and >15-fold, respectively. In other words, mechanochemistry of the dimers increases the amount of mechanical energy that a chain can absorb without fragmenting up to 15 times.

### Mechanochemistry and optical healing in bulk samples

**Fig. 5:**
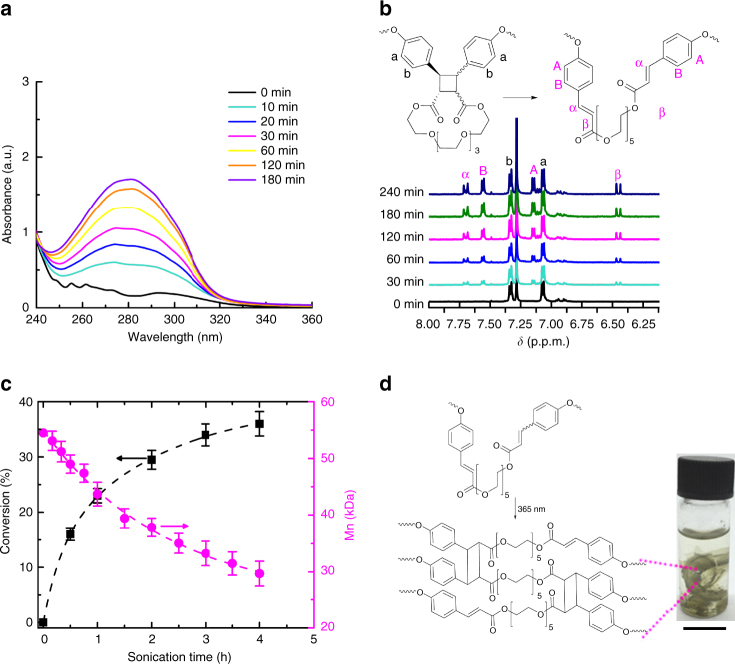
Summary of mechanochemistry of macrocyclic cinnamate dimers in solution. **a**, **b** The evolution of the UV-vis (**a**) and^1^H NMR (**b**) spectra of a solution of **P2** (*M*_n_ = 54 kDa) during sonication is consistent with dissociation of dimers **2a** to cinnamates, as evidenced by increases in the intensities of (**a**) the characteristic absorption band at 280 nm in UV and (**b**) ^1^H NMR resonances of the vinyl protons (marked α and β). **c** As is typical, sonication of **P2** resulted in competition between dimer dissociation (black curve and *y*-axis), which does not reduce the polymer size, and random chain fragmentation (magenta curve and *y*-axis), which does. Conversion % (black curve) was determined as (1 + *r*)^−1^, where *r* is the ratio of the intensities of the ^1^H NMR resonances marked b and B in Fig. 5b. This conversion % correlates well with the absorbance at 280 nm (Supplementary Fig. 17). Error bars are standard deviations. **d** Irradiating mechanically degraded **P2** creates an insoluble material, most likely by [2 + 2] photodimerization of cinnamates. The scale bar is 1 cm

We demonstrated mechanochemistry of macrocyclic dimers by sonicating solutions of **P2** (*M*_n_ = 54 kDa, PDI = 1.3) in tetrahydrofuran (at concentration of 4 mg mL^−1^ and bath temperature of 0–5 °C), following the standard approach^5,7–9,15,20,26,30,32,38,39^. The starting solution did not absorb above 250 nm (Fig. 5a), indicating the absence of the cinnamate chromophore. Sonication produced a broad absorbance band centered at 280 nm, whose intensity grew with sonication time. The appearance of this absorbance was accompanied by changes in the ^1^H NMR spectrum of the sample, with the intensities of peaks at chemical shifts characteristic of the cinnamate moiety (α, β, A and B, Fig. 5b) increasing relative to those of the intact dimers (marked as a and b in Fig. 5b) during sonication. Under identical conditions sonicating low molar-mass polymer **P2s** (*M*_n_ = 12 kDa, PDI = 1.4) didn’t affect its molar-mass distribution or composition (Supplementary Fig. 16). This lack of reactivity in the shorter polymer supports the mechanochemical rather than purely thermal or sonolytical mechanism of dimer dissociation in sonicated solutions of **P2**, because polymer length is known to correlate inversely with the chain susceptibility to hydrodynamic stretching during sonication^2,5,32^.

Sonication of polymers results in both mechanophore-based chemistry and non-selective backbone fragmentation^32^, which reduces the average molar mass of the polymer sample (Fig. 5c and Supplementary Fig. 18). Such competing selective and non-selective mechanochemical bond scission is thought to mimic the response of polymer chains in bulk materials under mechanical load^34,44,52^. Reuse of a degraded material requires restoration of its mechanical properties^35^, which can be achieved by reforming some of the lost load-bearing bonds. To demonstrate optical regeneration of load-bearing bonds in mechanically degraded samples of **P2**, we sonicated a solution of **P2** for 4 h to dissociate 36% of the dimers and to approximately halve its number-average molar mass. After removing the solvent, we split the degraded polymer in two parts, one of which was irradiated by 365 nm light (1.68 W cm^−2^) for 3 h. The untreated solid remained soluble in tetrahydrofuran whereas the irradiated one was partially insoluble in tetrahydrofuran, acetone, dimethylformamide or dichloromethane (Fig. 5d and Supplementary Fig. 19), suggesting mechanochemically generated cinnamates in the polymer backbones underwent photochemical [2 + 2] cycloaddition to form cyclobutane cross-links. This conclusion is supported by the appearance upon sonication of **P2** of an IR resonance at 1640 cm^−1^, which was previously associated with the cinnamate moiety^29,62,63^, and its disappearance upon irradiation of the sonicated sample at 365 nm, suggestive of photochemical dimerization of mechanochemically generated cinnamates (Supplementary Fig. 20). Changes in the mechanical properties of films of **P2** before and after sonication, and after sonication and optical healing, measured under macroscopic uniaxial tensile loading, are also consistent with mechanochemical dissociation of the dimers and their subsequent photodimerization. Thus, sonication of **P2** reduced its elastic modulus and ultimate strength 1.5 ± 0.2 and 3.1 ± 0.5-fold, respectively (Supplementary Fig. 21 and Supplementary Table 2), which is an expected outcome of backbone scission. In contrast, subsequent UV irradiation increased these parameters 3.9 ± 0.2 and 4.8 ± 0.7-fold, respectively. These results illustrate that the macrocyclic dimers allow optical regeneration of mechanically damaged volumes of polymers even when the damage is caused by fragmentation of backbones.

Quantitative characterization of mechanochemical reactivity in bulk polymers remains a largely unsolved challenge^32^, but qualitatively, the changes observed upon repeated uniaxial compression of films of **P2** to 550 MPa are consistent with load-induced dissociation of the cinnamate dimers. Macroscopic uniaxial compressive load is known to cause tensile loading of a subset of polymer chains^5,64^. First, loading films of **P2** produced a new absorption peak centered at 280 nm (Supplementary Fig. 22a), similarly to that observed upon sonication of **P2** in solution, indicative of the cinnamate chromophore. The peak intensity suggests that 5% of dimers dissociated in loaded samples. Second, solution^1^H NMR spectra of **P2** after loading manifest new set of signals suggestive of the cinnamate moiety (Supplementary Fig. 22b). Third, irradiating loaded samples at 365 nm for 4 h produced material that was partially insoluble in common solvents, indicative of photochemical cross-linking of the cinnamates produced by mechanical load (Supplementary Fig. 23). As expected, sonicating a suspension of this insoluble material did not solubilize the material or generated any detectable solutes because of the differences in the effect of ultrasound cavitation on a polymer solute and solid suspensions^23^.

## Discussion

The development of practical multi-modal stress-responsive polymers requires molecular architectures that are sufficiently flexible to allow tuning of mechanochemical kinetics and mechanical properties by chemical modifications of the mechanophores. Our computations suggest that the hidden length of the macrocyclic dimers is a simple design parameter to vary single-chain extensibility and toughness, although the effects of the macrocyclic size on the synthetic yield, solubility and processibility of the material remain to be fully quantified. The strong dependence of the kinetic stability of cyclobutane derivatives on relative orientations of the substituents (Fig. 2 and Supplementary Figs. 1–3, 5, and 6) means that the threshold single-chain force at which the dimers dissociate at useful rates is tunable over >1 nN by selecting the proper stereoisomer. Alternatively, using a mixture of stereoisomers in the same material may allow the range of macroscopic loads at which the dimers dissociate to be broadened. Although simple cinnamates have been used for optical detection of mechanical damage in polymers^36^, thanks to their fluorescent emission at ~ 450 nm, their near-UV absorption limits their mechanochromic applications. The capacity to tune systematically the absorption maximum of the phenylacrylate core over 280–370 nm by peripheral substitution at the phenyls is well documented^65,66^, as is photodimerization of such chromophores^54,67,68^. We see no obstacles of applying the synthetic and measurement approach described above to these derivatives. Several examples of even larger bathochromic shifts (up to 500 nm)^67,68^, and [2 + 2] photocycloaddition of such chromophores are known^69^. This literature suggests that at a minimum a series of analogs of **P2** with mechanochromic response covering the 300–400 nm (absorption) and 450–550 nm (emission) range is likely to be readily accessible and extending these ranges by an additional 100 nm seems plausible with already-described phenylacrylate derivatives. The data presented in this paper enables others to pursue such materials in parallel with our own ongoing effort.

In summary, we have demonstrated that macrocyclic dimers of cinnamate derivatives combine several modes of productive chemical response to mechanical load, making them promising candidates for the preparation of multi-modal stress-responsive polymers^52,70^. When macrochains of the dimers are stretched above 1 nN, fast dimer dissociation more than doubles the chain contour length and increases up to 15-fold the amount of energy that the chain can absorb without backbone fragmentation (Fig. 4). This dissociation produces the cinnamate chromophore^36,66^ that could allow optical detection of volumes of the material that had experienced sufficient local loads to have been weakened by loss of load-bearing bonds. These regions can be reinforced (Supplementary Fig. 21) by light to yield cyclobutane cross-links by photochemical dimerization of cinnamates. Our quantum-chemical calculations and single-molecule force experiments reveal how structural parameters of the dimers, including the position and the length of the hidden length linker and the relative orientation of the substituents affect the mechanochemical reactivity of the dimer and establish quantitatively how these parameters determine single-chain micromechanics, including single-chain extensibility and toughness (Fig. 2 and Supplementary Figs. 2, 5, 6, and 14). As such, this data constitutes the most detailed characterization reported to date of the effect of applied force on the kinetics and mechanism of dissociation of the increasingly popular cyclobutane core^8,9,13,26,37,39^. Because mechanochemical kinetics, single-chain extensibility and toughness of the dimers and their polymers are tunable by simple synthetic modifications, the quantitative structure/property correlations presented above facilitate straightforward identification of candidate cinnamate dimers to yield polymers with a range of stress-responsive behavior.

## Methods

### Synthesis of **2a**

(1R,20S,21R,22S)- and (1R,20R,21S,22S)-21,22-bis(4-hydroxyphenyl)-3,6,9,12,15,18-hexaoxabicyclo[18.2.0]docosane-2,19-dione: Under N_2_, a solution of ethane-1,2-diyl (2E,2′E)-bis(3-(4-hydroxyphenyl)acrylate) (Supplementary Figs. 8, 24–27) (1.06 g, 2 mmol, 1 equiv.), benzophenone (3.644 g, 20 mmol, 10 equiv.) and KClO_4_ (1.38 g, 10 mmol, 5 equiv.) in acetonitrile (200 mL) was irradiated at 365 nm for 48 h at room temperature. The solution was concentrated and the crude product was purified with column chromatography (CH_2_Cl_2_/acetone = 5:3) to give the mixture of syn-**2a** and anti-**2a** (0.47 g) as a white solid in 47% yield (the molar ratio of 1 syn to 5.2 anti was determined by integrating the^1^H doublets at 6.55 and 7.16 p.p.m., respectively, Supplementary Fig. 28).^1^H NMR (CDCl_3_, 500 MHz): δ(p.p.m.) = 7.15–7.13 (d, *J* = 8.4 Hz, 2H), 6.77–6.75 (d, *J* = 8.4 Hz, 2H), 5.16 (s, 1H), 4.44–4.40 (m, 1H), 4.19–4.15 (m, 2H), 3.76–3.62 (m, 8H), 3.43–3.41 (m, 1H). anti-**2a**)^13^C NMR (CDCl_3_, 500 MHz): δ(p.p.m.) = 173.09, 155.49, 132.42, 129.02, 128.07, 115.62, 70.77, 70.56, 70.39, 69.23, 64.54, 47.31, 44.31. syn-**2a**)^13^C NMR (CDCl_3_, 500 MHz): δ(p.p.m.) = 173.06, 155.29, 129.92, 128.07, 115.05, 70.68, 70.53, 70.28, 69.01, 64.31, 44.79, 43.18. MS (LC-MS) calculated for C_28_H_34_O_10_ *m*/*z*: 530, found *m*/*z* = 553.0 (M + 23).

### Polymerization of **2a** to **P2**

A purified mixture of syn-**2a** and anti-**2a** (300 mg, 0.566 mmol, 1 equiv.), glutaric acid (74.7 mg, 0.566 mmol, 1 equiv.), and dimethylaminopyridinium toluenesulfonate (67 mg, 0.14 mmol, 0.4 equiv.) were added to a 25 mL flask. Dry CH_2_Cl_2_ (5 mL) was added by syringe, and the suspension was heated to 37 °C while stirring until the solution became homogenous. After cooling to room temperature, *N*,*N*-diisopropylcarbodiimide (0.265 mL, 1.7 mmol, 3 equiv.) was added dropwise by syringe. The mixture was stirred for 96 h, followed by the addition glutaric acid (18.6 mg, 0.141 mmol, 0.25 equiv.) and subsequently *N*,*N*-diisopropylcarbodiimide (0.066 mL, 0.42 mmol, 0.75 equiv.) and the solution was stirred for another 24 h. The mixture was dissolved in CH_2_Cl_2_ and poured into methanol to precipitate **P2** as a white solid (340 mg, 91% yield).^1^H NMR (CDCl_3_, 500 MHz): δ(p.p.m.) = 7.31–7.30 (d, J = 8.6 Hz, 4H), 7.05–7.03 (d, J = 8.2 Hz, 4H), 4.44–4.40 (m, 4H), 4.20–4.17 (m, 2H), 3.78–3.62 (m, 16H), 3.47–3.45 (m, 2H), 2.72–2.67 (m, 4H), 2.18–2.15 (m, 2H). *M*_n_(GPC) = 54,000 g/mol, *M*_w_ = 67,000 g/mol, PDI = 1.3 (Supplementary Fig. 10).

### Single-molecule force experiments

Cleaned AFM tips and silicon wafers (substrate) were silanized by placing them in the atmosphere of aminopropyldimethylmethoxysilane in a dry N_2_-purged desiccator for 1.5 h at 25 °C. Immediately after being taken out, the silanized tips were rinsed three times with methanol and then placed in a 110 °C oven for 10 min for activation. **P2** was anchored to the wafers by placing a 200 μL drop of a DMF solution of **P2** (0.5 μmol mL^−1^) and DCC (0.75 μmol mL^−1^) onto the substrate for 30 min at room temperature in the dark to allow the carboxy groups at one end of **P2** to react with the amine groups of the wafer-bound silane, leaving free the carboxy group at the other terminus of each polyester chain for subsequent binding to silanized AFM tips. After 30 min, the wafers were rinsed carefully with DMF to remove any unanchored molecules.

### Ultrasound sonication experiments

The experiments were performed on a Vibra Cell 505 liquid processor with a 12.8 mm (diameter) titanium solid probe (Sonics Materials) using THF solutions of polymers at 4 mg mL^−1^ in a 3-neck glass cell immersed in an ice bath. Prior to sonication, the solution was sparged with N_2_ for 20 min. Sonication was performed under N_2_ with a pulse sequence of 1 s on followed by 1 s off at a nominal power of 8.7 W cm^−2^. The temperature of the solution was maintained at 0–5 °C. Aliquots of 0.5 mL were removed periodically from the cell for GPC,^1^H NMR and UV-Vis analyses. For GPC analysis, the aliquotes were diluted to 0.8 mg mL^−1^. For^1^H NMR measurements, the polymers were precipitated with methanol and redissolved in CDCl_3_ at ~ 25 mg mL^−1^. IR spectra were measured with Nicolet iS10 FT-IR spectrometer on thin films.

### Mechanical experiments

Films of intact **P2** were cast from CH_2_Cl_2_ solutions (40 mg mL^−1^). Films of sonicated **P2** were cast from a solution obtained by concentrating a THF solution of **P2** that was sonicated for 4 h as described in the preceding section. Films of sonicated and UV irradiated material were obtained by irradiating films of sonicated **P2** at 365 nm 1.68 W cm^−2^ incident power for 4 h. All films were 0.3 mm thick and cut into rectangular strips (8.0 × 1.3 × 0.3 mm) for tensile testing, which was performed on an Instron 3343 tensile tester. Strips were directly mounted on the grips and the initial strain rate of 0.02 s^−1^ was applied. For compression test pristine **P2** was molded into circular films with diameter of 7 mm and thickness of 0.5 mm. The films were load into a pellet press, manually compressed by a laboratory press to 550 MPa and maintained at this compressive load for 5 min. This procedure was repeated 5 times for each film and some of the compressed films were further irradiated by 365 nm UV light for 4 h.

### Calculation of single-chain force/extension curves of P2

No analytical solution describes the force/extension curve or the evolution of the composition of the chain during a dynamic single-molecule force experiment^34,46^. Consequently, force/extension curves (that underlie the statistics in Fig. 4b, d) were calculated by incrementing stretching time, *t* (and consequently the controlled extension, *L* = *tv*, where *v* is the stretching rate of 1 μm s^−1^) and calculating all other parameters determining the behavior of a given chain (chain length; its restoring force; its composition; bending force of the cantilever, and the survival probability of each dimer in the chain, *s*) for each sequential value of *t* and *L* (Supplementary Fig. 29). Force/extension curves resulting from dissociation of syn dimers were calculated independently of those for anti dimers (in the former case, the simulation stopped when the final syn dimer in the initial chain dissociated; in the latter case, the simulations started with a copolymer of *EE* diene (Fig. 4a) and anti dimer). Because isomerization of each monomer in a stretched chain is a stochastic process, for a chain containing *n* dimmers, we calculated ~ 10^5^ *n* ^3/2^ (or 10^8^, whichever was smaller) individual force/extension curves. The values of *n* for which the curves were calculated matched those for the set of chains studied by the SMFS. In each simulation a unique combination of *n* monotonically decreasing random numbers from 0.9999 to 10^−4^ (so called survival probability vector, *S*) was used to define at which simulation step each dimer dissociated. These sets of random numbers were generated and converted to the total relative probability that each calculated force/extension curve would appear among all generated curves using Supplementary Eq. 1. In calculating the distribution of single-chain forces for dissociation of anti dimers for comparison with the experiment, only the dissociation of the first *m* dimers was considered, where *m* is the number of experimentally observed dissociations.

### Data availability

The data sets generated during and/or analyzed during the current study are available from the corresponding authors on reasonable request.

## Electronic supplementary material

Supplementary Information
