# Supplementary material for: Multi-modal mechanophores based on cinnamate dimers
Source: Nat Commun. 2017 Oct 27;8:1147. doi: 10.1038/s41467-017-01412-8 (PMC5660084; doi:10.1038/s41467-017-01412-8)
Supplement: Supplementary file 1 — Supplementary Information [file 41467_2017_1412_MOESM1_ESM.pdf]

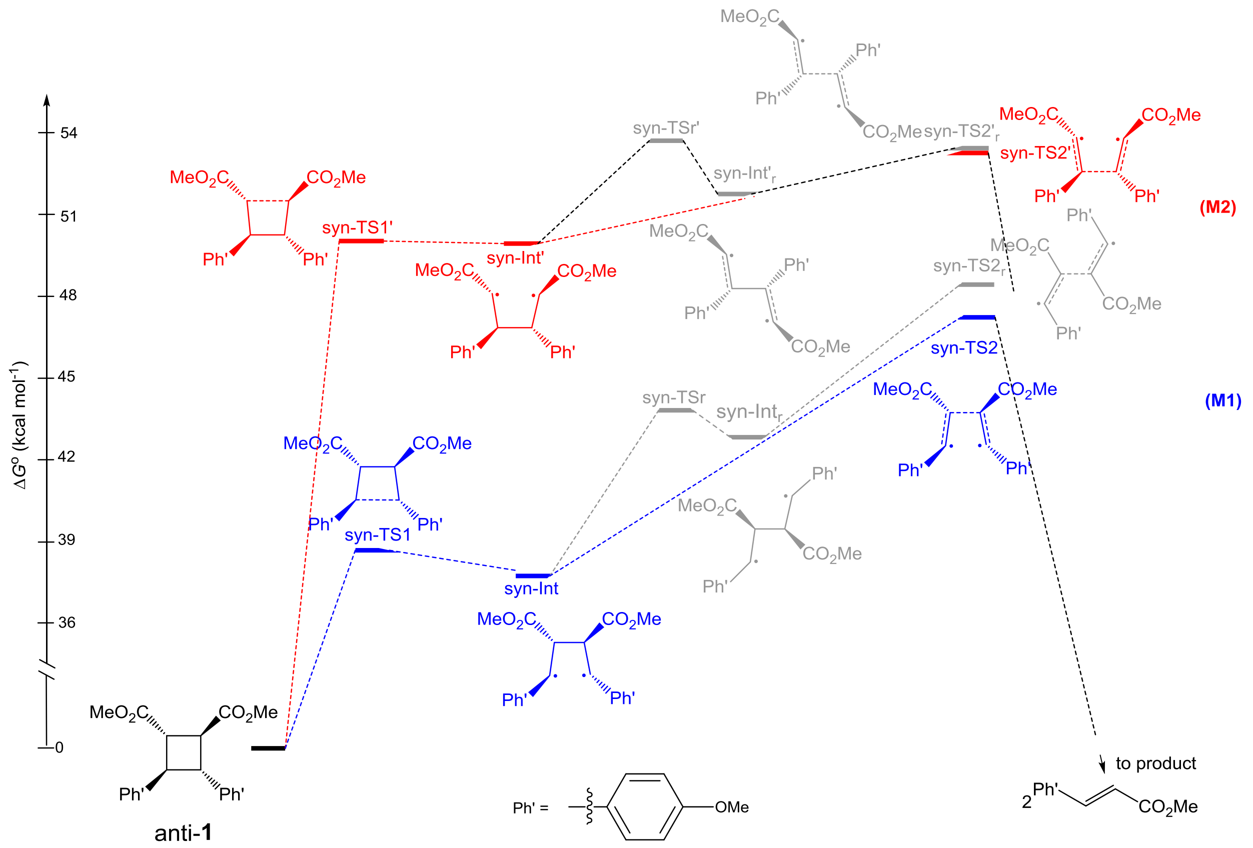


**Supplementary Figure 1**. **Computed mechanism of thermal dissociation of strain-free anti-1.** Grey structures correspond to the alternative reaction path that becomes important only under force (Supplementary Figs. 3c-d). In the absence of force, **M1** (blue) dominates. All calculated at the uMPW1K/6-31+G(d) level.

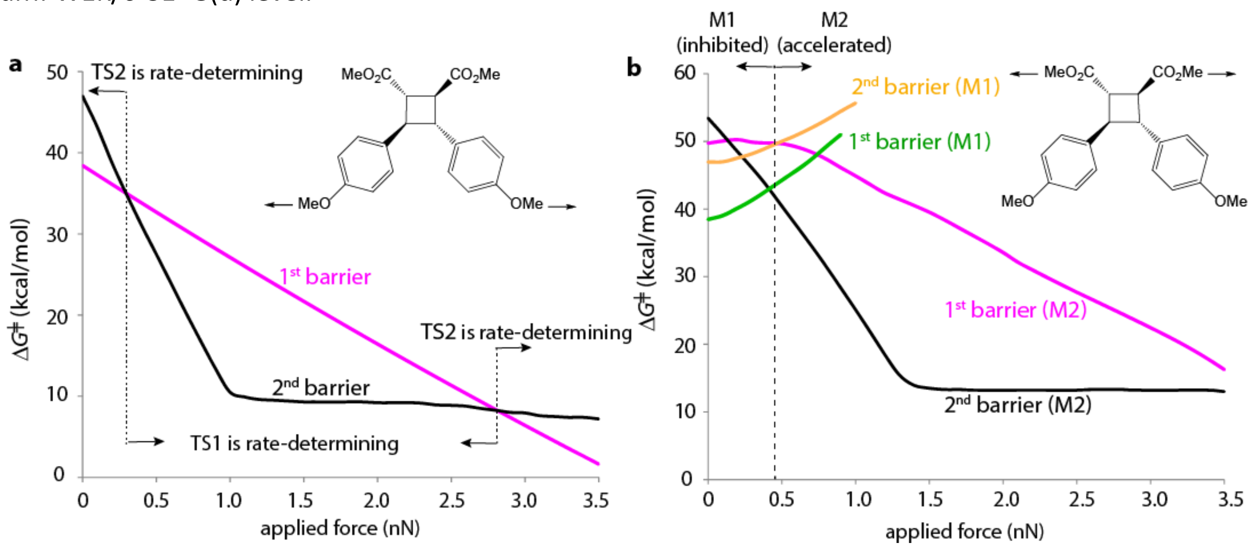


**Supplementary Figure 2. Calculated force-dependent free energies barriers for dissociation of anti-1.** Force applied at (a) phenoxymethyl or (b) carboxymethyl groups. Below 0.97 nN (phenoxymethyl pull, a) or 1.45 nN (carboxymethyl pull, b) the energy of the 2$^{nd}$ barrier (**M1** in a and **M2** in b) is determined by the difference in the energy of the 2$^{nd}$ transition state and the reactant (Supplementary Fig. 1); above these forces, the energy of the 2$^{nd}$ barrier is determined by the energy difference between $Int_r$ and $TS2_r$ (Supplementary Figs. 3c-d).

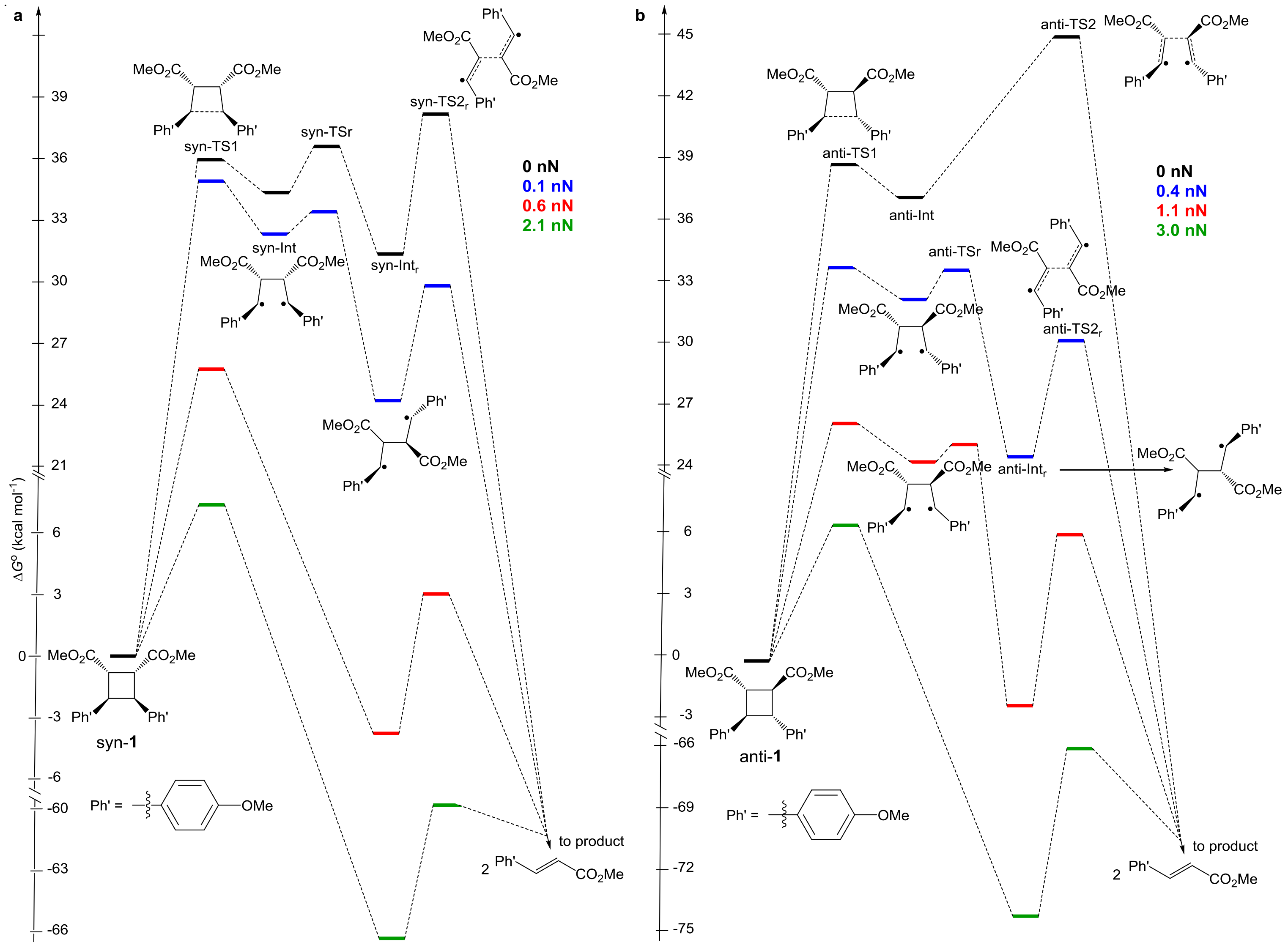


**Supplementary Figures 3a-b**. **Calculated dissociation mechanisms of 1**. Free energy profiles along the minimum-energy **M1** path for dissociation of syn-**1** (a) and anti-**1** (b) in the absence of force (black) and at different forces applied across the C atoms of the methoxyphenyl groups. The profiles highlight changes in the nature of the rate-determining transition state with force.

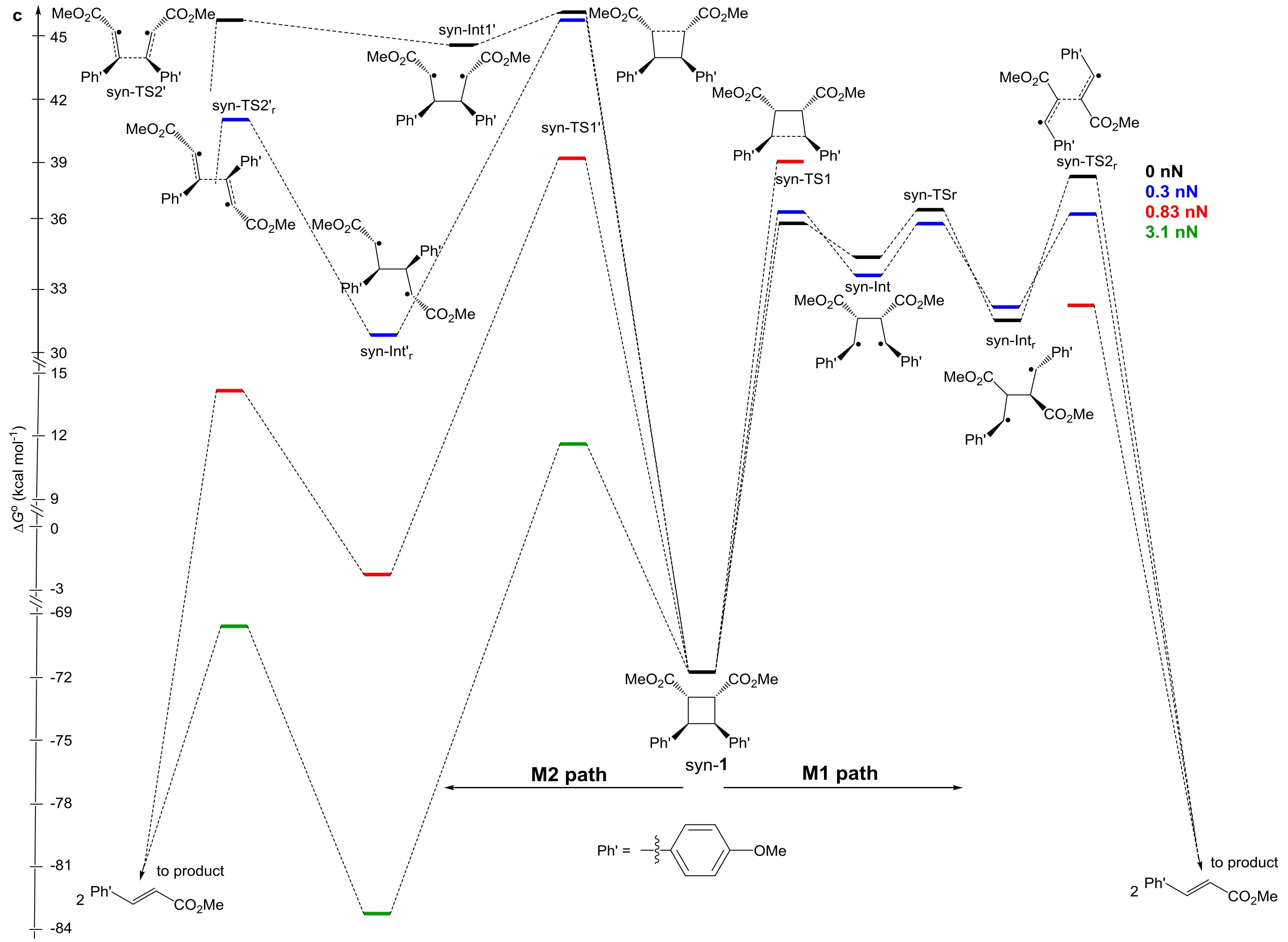


**Supplementary Figure 3c Calculated dissociation mechanisms of 1.** Free energy profiles along the **M1** and **M2** paths for dissociation of syn-**1** in the absence of force (black) and at different forces applied across the C atoms of the carboxymethyl groups. The profiles highlight changes in the nature of the rate-determining transition state with force.

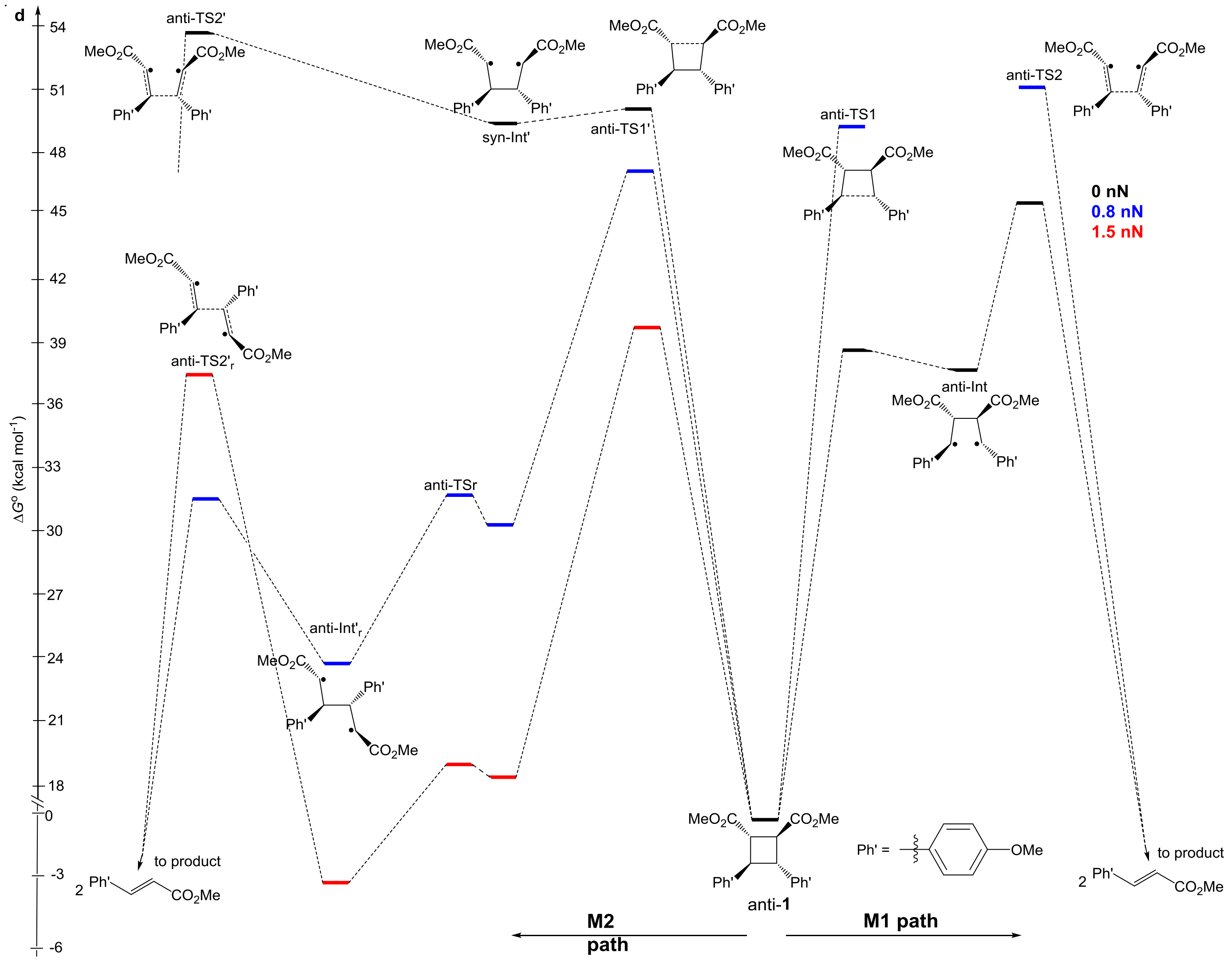


**Supplementary Figure 3d Calculated dissociation mechanisms of 1.** Free energy profiles along the **M1** and **M2** paths for dissociation of anti-**1** in the absence of force (black) and at different forces applied across the C atoms of the carboxymethyl groups. The profiles highlight changes in the nature of the rate-determining transition state with force. We didn't optimize the intermediate of **M1** at force >0.5 nN because it plays no kinetic role.

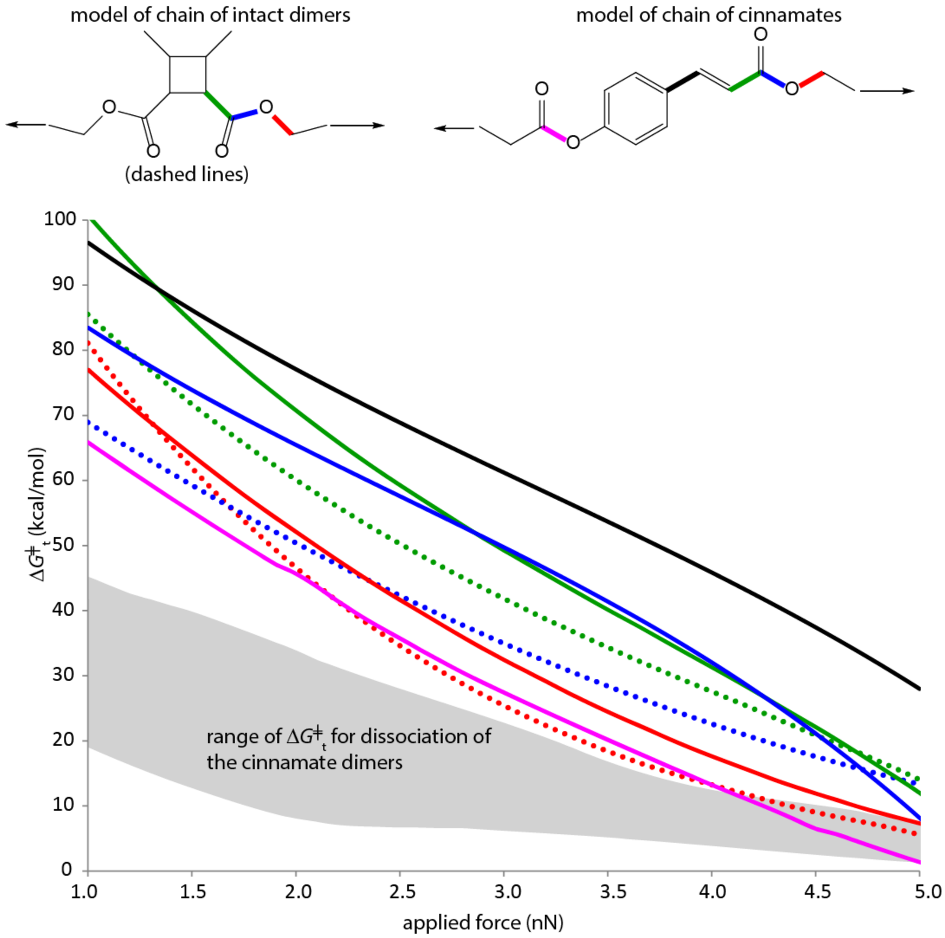


**Supplementary Figure 4**. **Site-specific vs. non-specific backbone dissociation**. Computed force-dependent free energies of activation of homolysis of backbone bonds in models of polymers of cinnamate dimers before and after dissociation of cinnamate dimers. Each line corresponds to $\Delta G^{\ddagger}$ for homolysis of the bond of the same color highlighted in bold: dashed lines refer to homolysis of bonds in diethyl (1R,2S,3R,4S)-3,4-dimethylcyclobutane-1,2-dicarboxylate (left structure), solid lines to ethyl *E*-3-(4-(propionyloxy)phenyl)acrylate (right structure). Force was applied to the C atoms of the Me groups (as illustrated by arrows). $\Delta G_t^{\ddagger}(f)$ for dissociation of the cinnamate dimers studied in this work are contained in the grey area. All calculations were at the uMPW1K/6-31+G* level in vacuum.

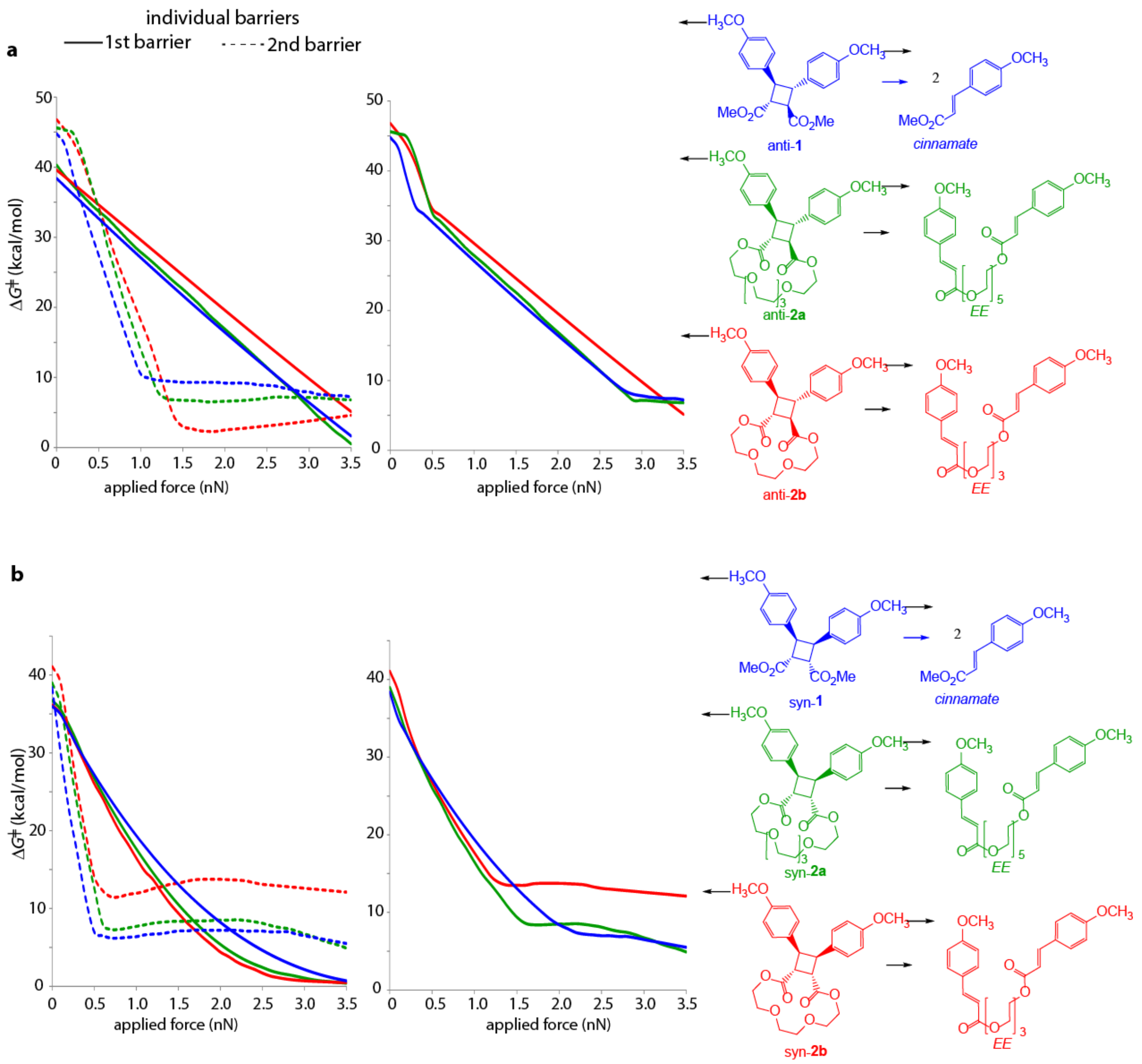


**Supplementary Figure 5. Force-dependent barriers of dissociation of 2.** Energies of individual kinetic barriers (left graphs, solid and dashed lines represent barriers to dissociation of the 1st and 2nd scissile bonds, respectively) and the total activation free energy of dissociation $\Delta G_t^{\ddagger}$ (right graphs) of anti-**2** (a) and syn-**2** (b) macrocycles vs. applied force. All dissociations proceed by the M1 mechanism (Fig. 1b and Supplementary Figs. 1, 3a-b). The graphs' colors match those of the reactions on the right. The pulling axes are defined by black arrows (force is applied at the C atoms of the Me groups).

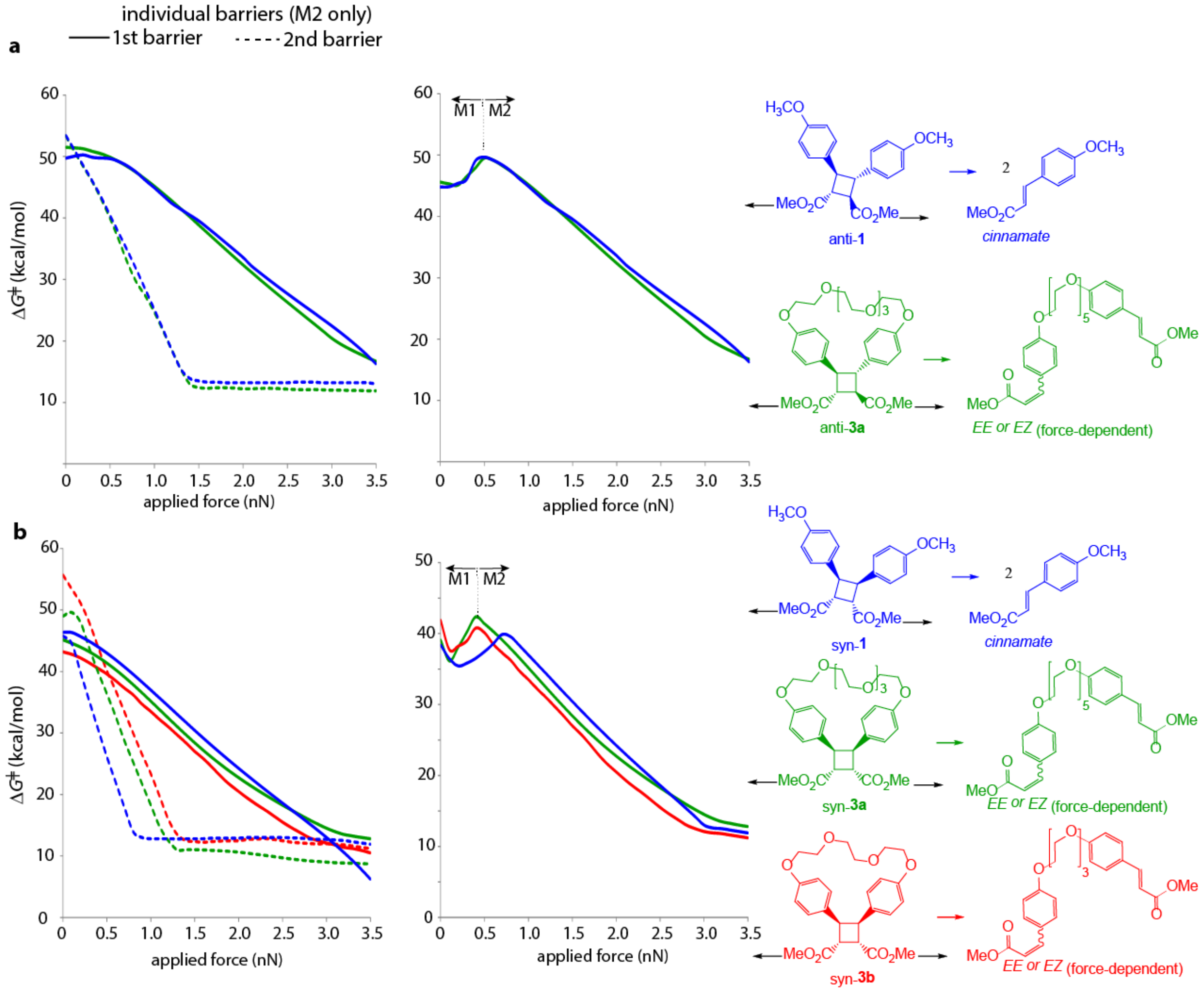


**Supplementary Figure 6. Force-dependent barriers of dissociation of 3.** Energies of individual kinetic barriers (left graphs, solid and dashed lines represent barriers to dissociation of the 1st and 2nd scissile bonds, respectively) of the **M2** mechanism and the total activation free energy of dissociation, $\Delta G_t^{\ddagger}$ (right graphs) of anti-**3** (a) and syn-**3** (b) macrocycles compared with the same energies for non-macrocyclic cinnamate dimers. Note that at force <0.5 nN (anti-**1**, anti-**3a** and syn-**3**) or <0.8 nN (syn-**1**) the dimers dissociate by **M1** mechanism whose barriers are omitted for clarity from the left-side graphs. Above these forces, the dissociation mechanism is **M2** (Fig. 1b and Supplementary Figs. 1, 3c-d). The graphs' colors match those of the reactions on the right. The pulling axes are defined by black arrows (force is applied at the C atoms of the Me groups). We didn't calculate the activation energies of anti-**3b** (n=1, Fig. 1a) because its reactant was calculated to be 32 kcal mol$^{-1}$ less stable than the syn-**3a** analog, making it synthetically inaccessible.

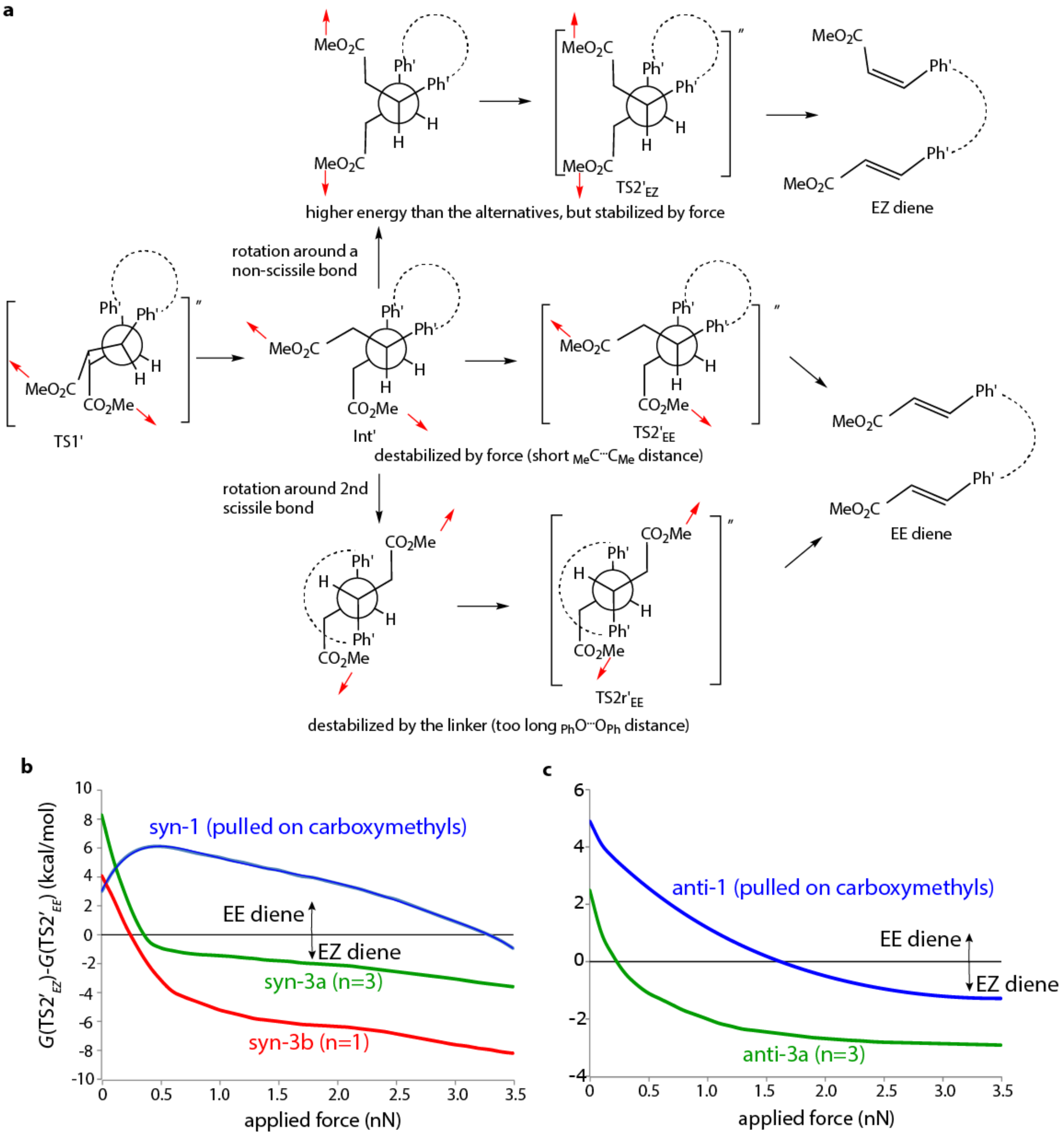


**Supplementary Figure 7. Mechanistic origin of isomerization of 3 during dissociation.** Mechanochemical dissociation of series 3 dimers produces either *EE* or *EZ* dienes, depending on the applied force and the length of the linker. (a) The relationship between various rotational conformers of Int' and TS2' along the **M2** dissociation path; the pulling axis is defined by the red arrows; Ph' = *p*-phenoxy. (b) The free energy of the $TS2'_{EZ}$ conformational ensemble (leading to EZ dienes) relative to the EE-generating transition states ($TS2'_{EE}$ + $TS2r'_{EE}$ in a). Negative values mean that the $TS2'_{EZ}$ is more stable than the $TS2'_{EE}$ analogs with the dissociation producing *EZ* dienes. Mechanochemical dissociation of series 3 dimers is calculated to produce *EZ* dienes over practically relevant forces (force is applied at the C atoms of the Me groups).

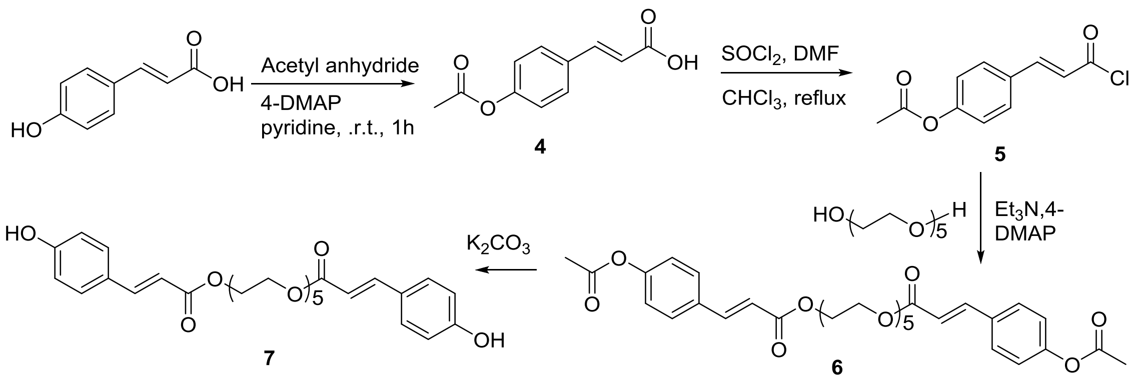


**Supplementary Figure 8. Synthesis of precursors to 2a.**

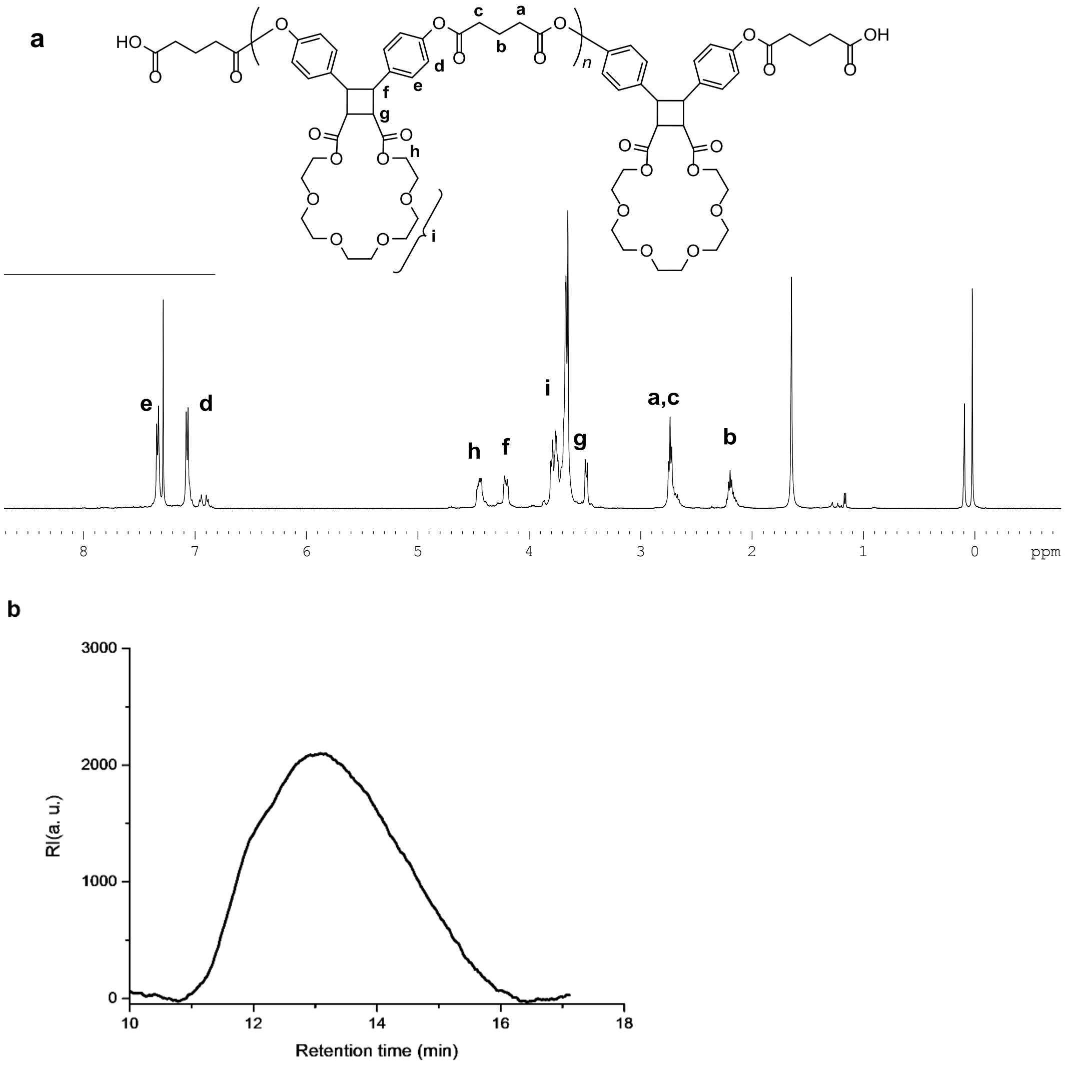


**Supplementary Figure 9. Spectroscopic characterization of P2**. (a) $^{1}$H NMR; (b) GPC.

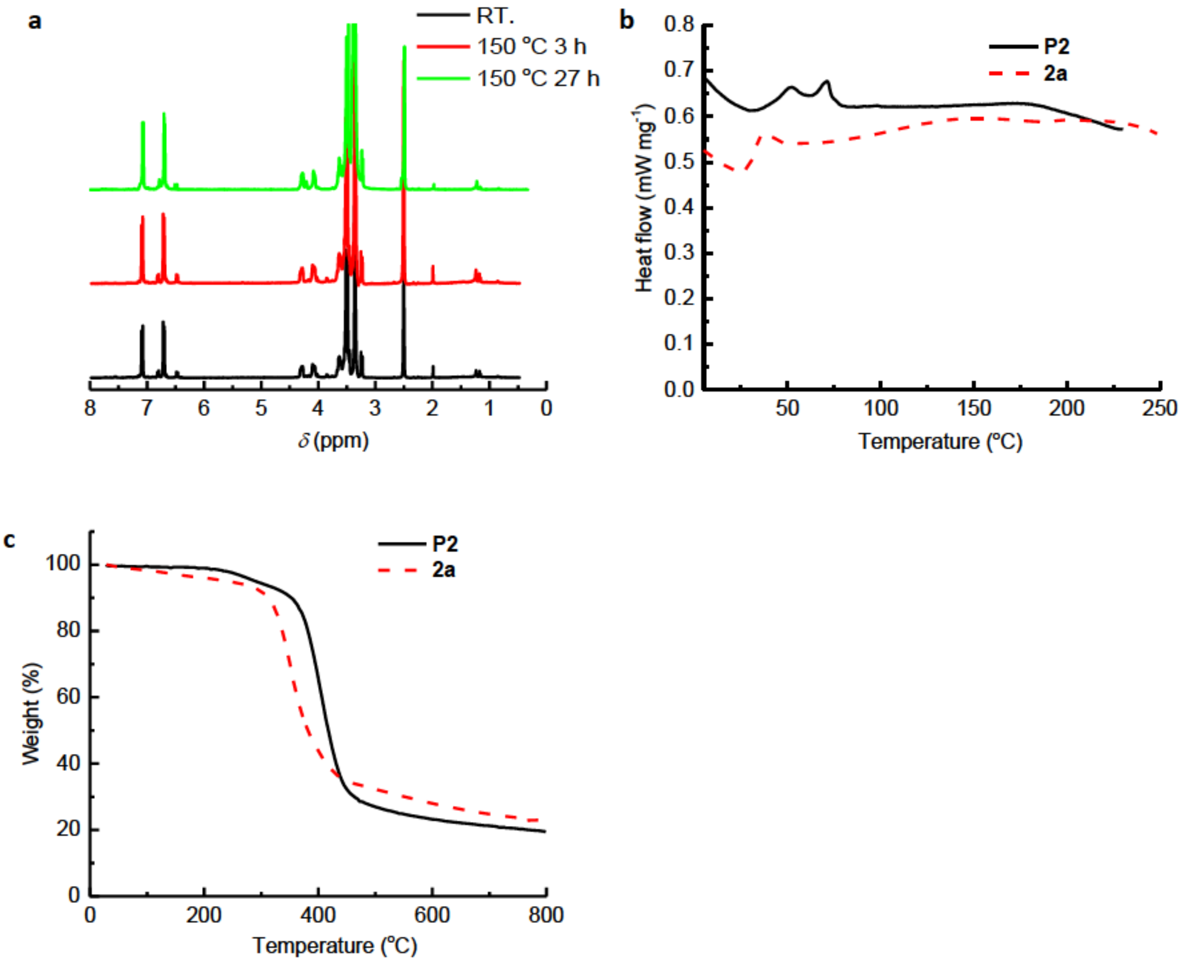


**Supplementary Figure 10. The evidence of high thermal stability of cinnamate dimers.** (a) $^1$H NMR spectra of a solution **2a** in DMSO-$d_6$ heated under vacuum for up to 27 h: the spectra show no new peaks or changes in the relative intensities of the original peaks; (b) DSC measurements on **P2** and **2a** under $N_2$ (heating rate 10 °C/min) show no evidence of chemical reaction[1, 2] up to 250 °C. The peak at 45 °C (**2a**) or 55 °C (**P2**) is typical of materials containing crown-either moieties (http://www.chemicalbook.com/ChemicalProductProperty_EN_CB4445218.htm) and the peak at 70 °C in **P2** is typically attributed to melting of crystalline domains of polyesters.[3, 4] (c) thermogravimetry of **P2** and **2a** (heating rate 10 °C/min) shows only pyrolysis at >400 °C.[5, 6, 7]

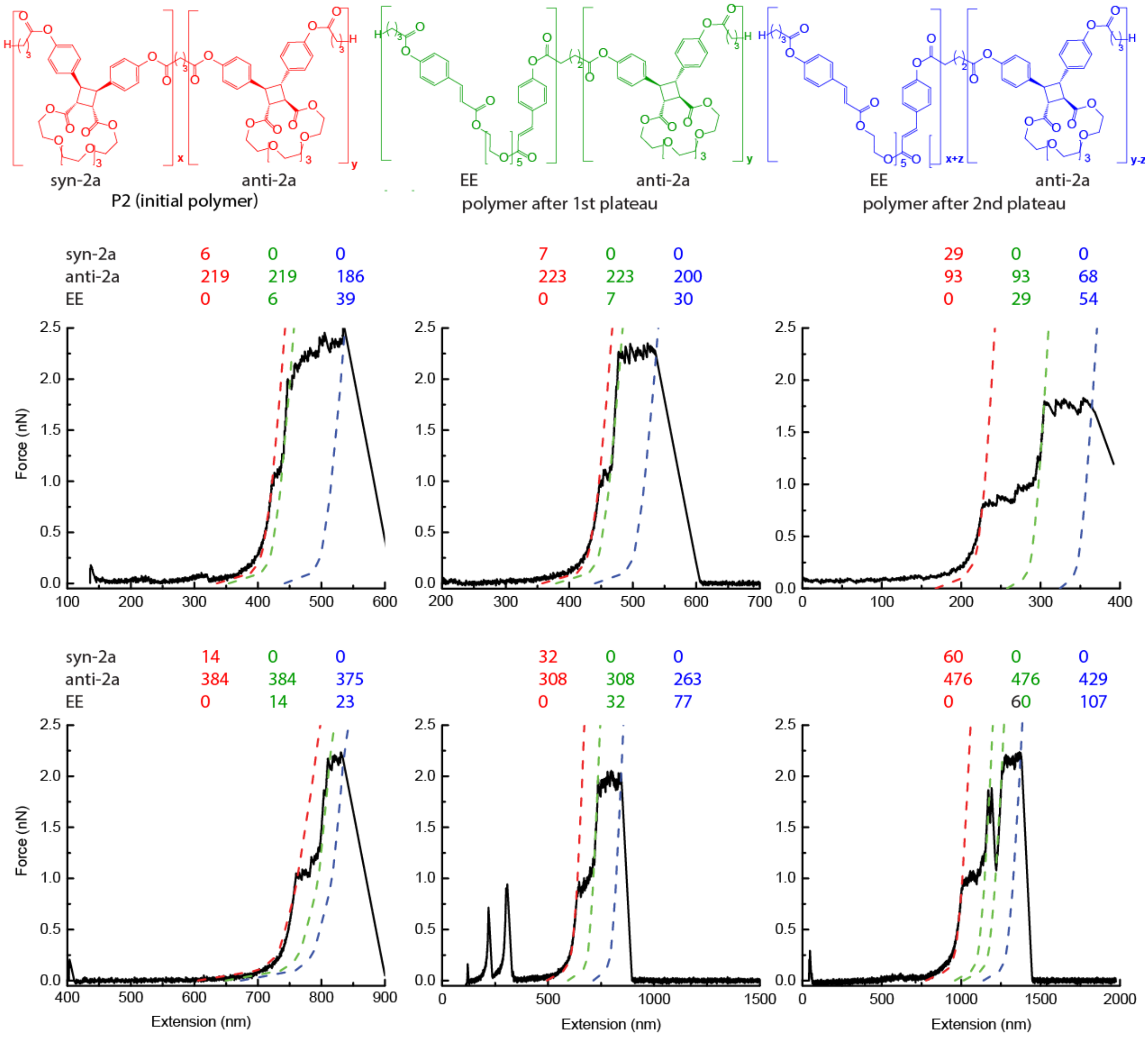


**Supplementary Figure 11. Representative force/extension curves for copolymer P2**. Dashed lines are calculated force/extension curves for chains of the composition shown above each group; the numbers have the same color as the lines to which they refer. For illustrative purposes the calculated force/extension curves for polymers containing syn or anti dimers are shown beyond the force at which they dissociated under the experimental conditions.

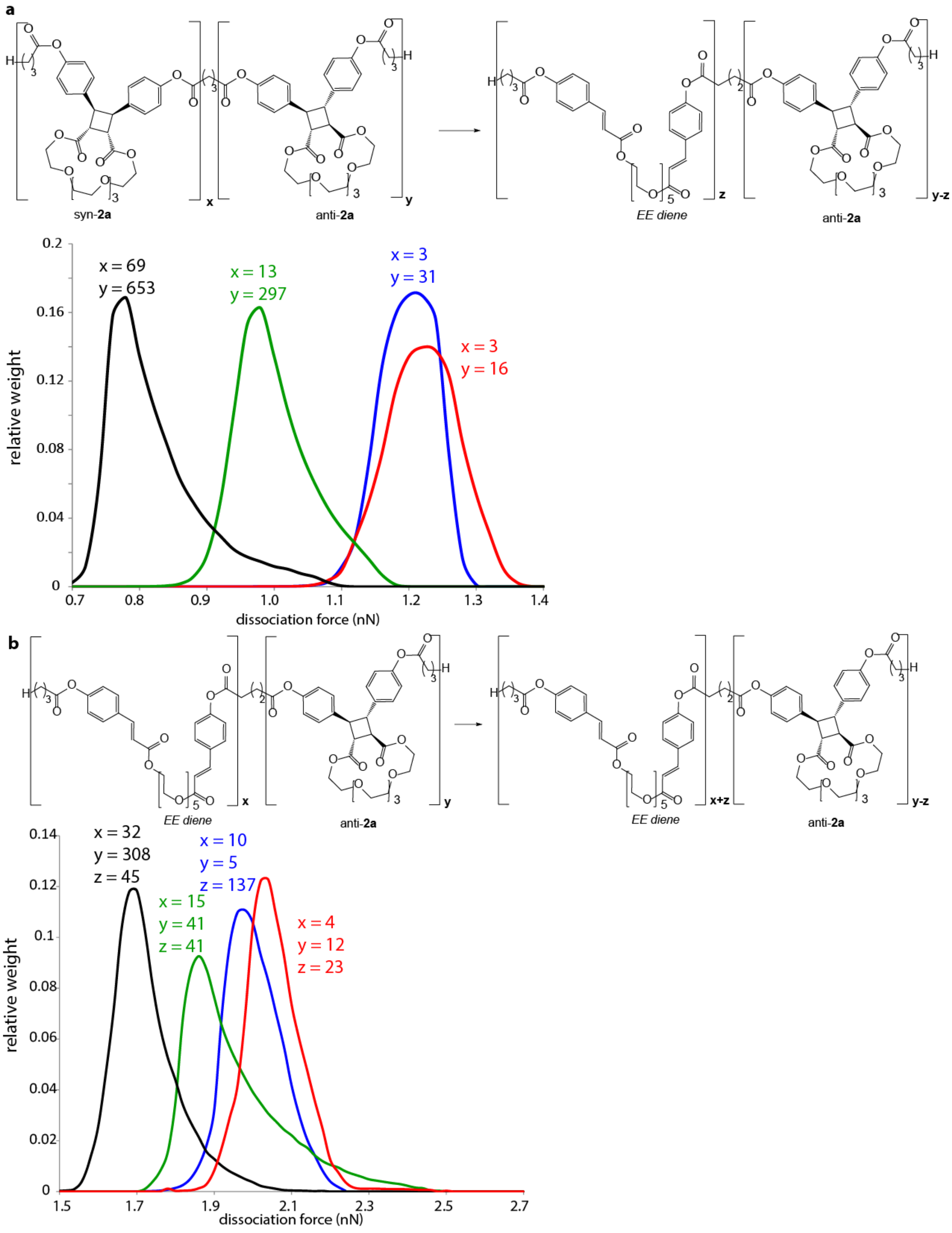


**Supplementary Figure 12. Calculated distribution of SMFS dissociation forces.** (a) dissociation of only syn-**2a** monomers and (b) dissociation of anti-**2a** monomers. To facilitate comparison, all distributions were normalized to the same area; in calculating the distributions in Fig. 4b, the distributions of individual macrochains had relative contributions proportional to the number of corresponding dimers in each chain (indicated by *x* and *y* for syn and anti dimers, respectively).

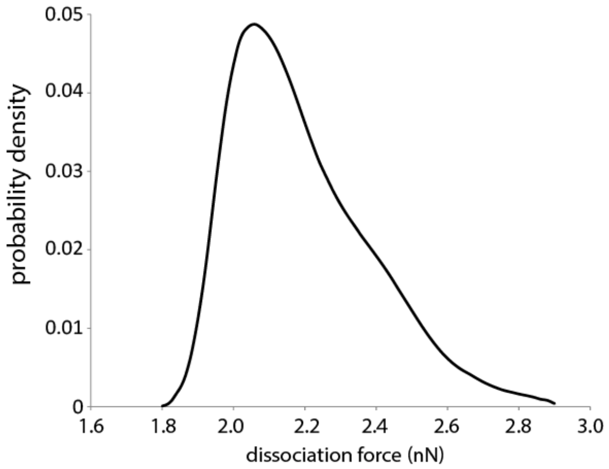


**Supplementary Figure 13. Effect of chain detachment on the dissociation force distribution**. Calculated distribution of dissociation forces of anti dimers (2nd plateau) for the 30 experimentally studied macromolecular fragments in the absence of detachment.

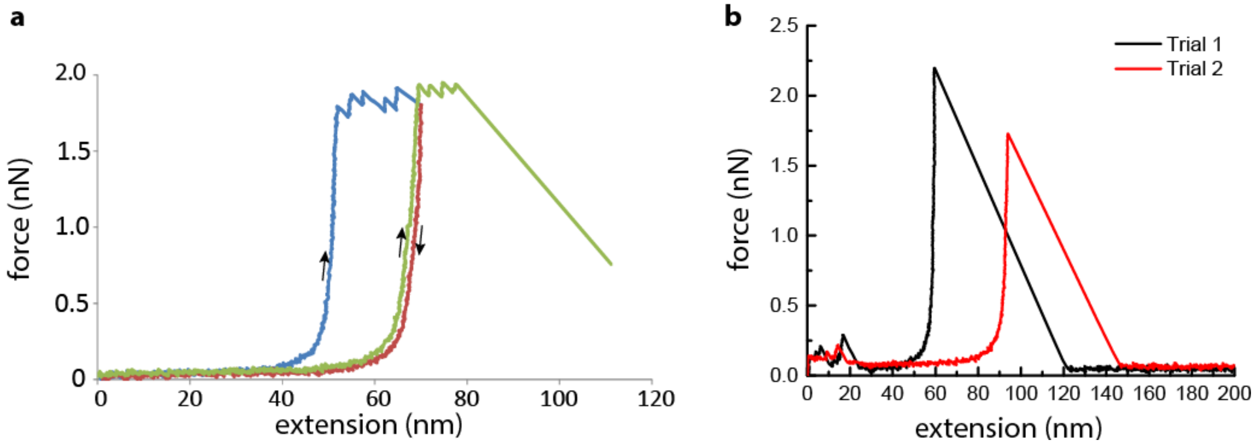


**Supplementary Figure 14**. **The plateaus are only observed in chains containing dimers.** (a) Traversing the plateau results in an irreversible elongation of the chain contour length because reversing the direction of translation of the AFM tip (blue: retraction followed by red, approach) does not retrace the original force/extension curve(blue) but follows a new force/extension relationship corresponding to a longer chain. The similarity of the green and red curves below 1.8 nN indicates that if the force is too low to induce mechanochemical dissociation, stretching of the chain is fully reversible. (b) stretching macromolecules containing cinnamate moieties but not the dimers (polyester derived from **7**, Supplementary Fig. 8) doesn't produce a plateau.

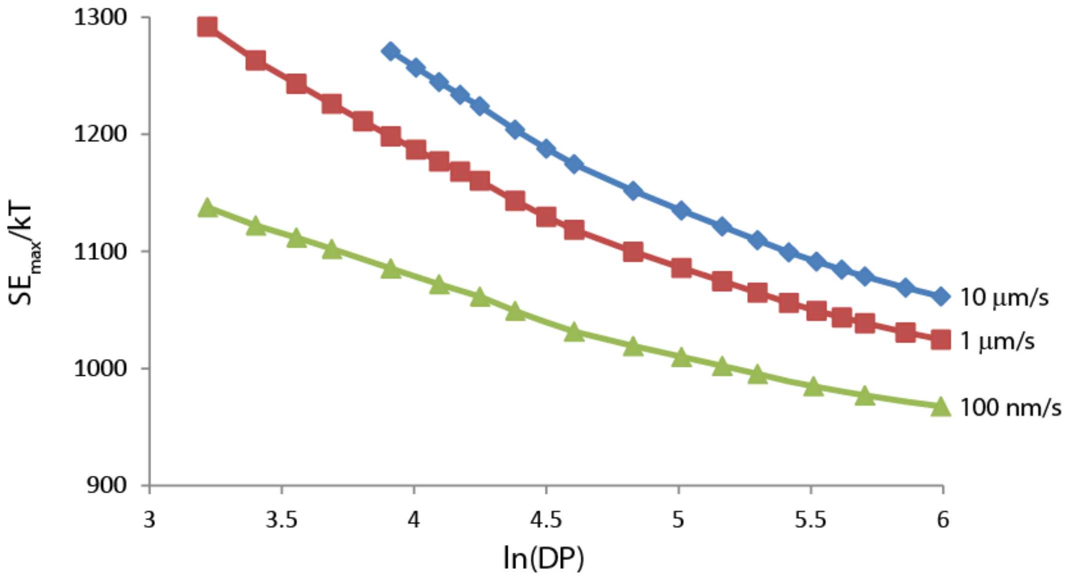


**Supplementary Figure 15. Calculated most-probable single-chain strain energy before detachment**. $SE_{max}$ in units of thermal energy (kT, where k is Boltzmann constant and T is the temperature) per monomer as a function of the degree of polymerization (DP) for poly(anti-**2a**) stretched at 3 different rates. We do not know the chemistry responsible for the chain detachment, which may proceed by multiple mechanisms, and we modeled this detachment as a thermally-activated process that is accelerated 5-fold per 0.1 nN of applied force, resulting in the detachment probability of $\leq 10^{-3}$ at 2 nN increasing to $\geq 0.999$ at 2.5 nN (exact numbers depend on the fragment length; see the Supplementary Methods below for further details).

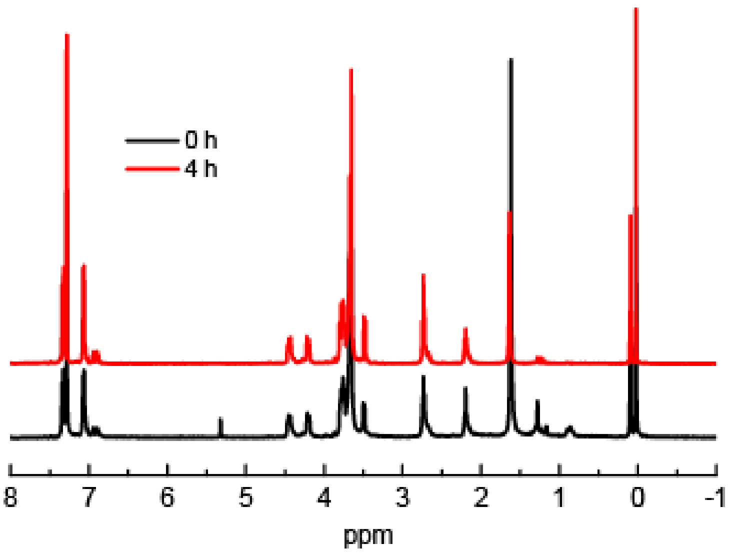


**Supplementary Figure 16. Short analogs of P2 are mechanochemically inactive**. $^{1}$H NMR spectra of a solution of short copolymer of syn-**2a** and anti-**2a P2s** ($M_n$ = 12 kDa) before sonication (black) and after 4 h of sonication (red).

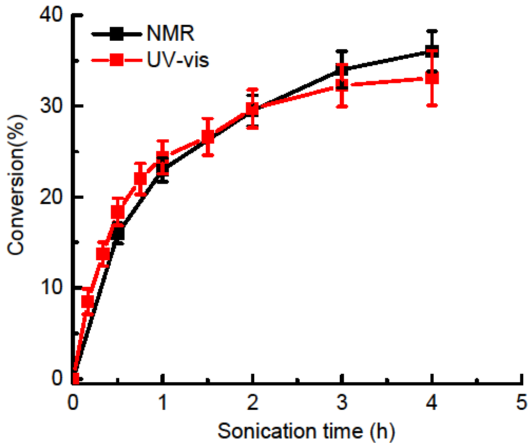


**Supplementary Figure 17**. **Consistent degree of conversion by two methods**. Comparison of the fraction of dimers that dissociated during sonication of **P2** as a function of the sonication time determined from $^{1}$H NMR (as described in the caption for Fig. 5, main text) and absorption intensity at 280 nm using the measured extinction coefficient of cinnamate ester of 23800 $M^{-1}$ $cm^{-1}$ (the reported[8] value is 23000 $M^{-1}$ $cm^{-1}$).

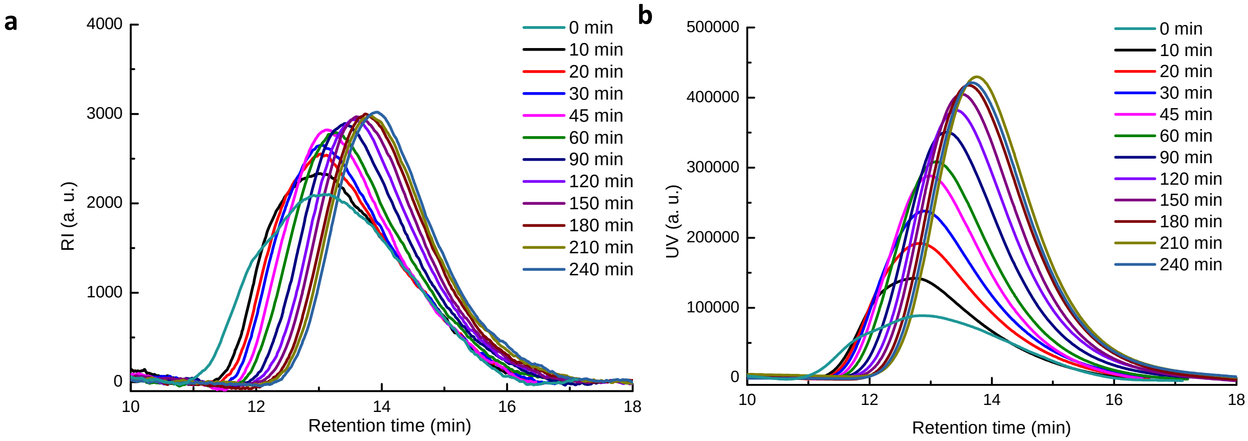


**Supplementary Figure 18. Evidence of dimer and backbone dissociation during sonication**. Gel-permeation chromatograms of a solution of **P2** as a function of sonication time: (a) refractive-index detector; (b) absorption at 280 nm.

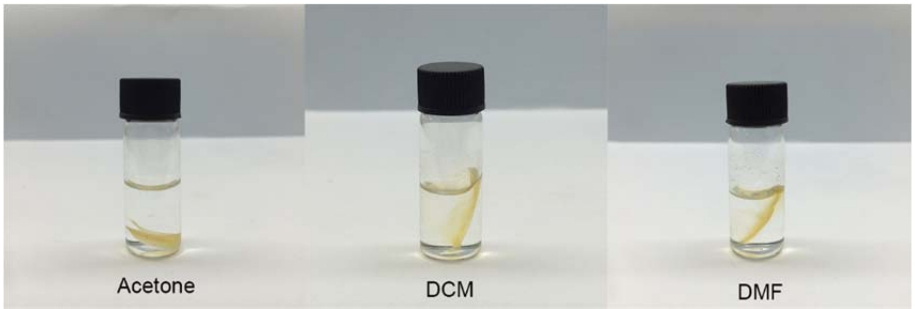


**Supplementary Figure 19. Optical healing yields insoluble material**. After irradiation, the sonicated and mechanically degraded **P2** was partially insoluble in acetone, DCM and DMF, suggesting [2 + 2] photodimerization of cinnamates. Irradiation of **P2** prior to sonication doesn't change its solubility.

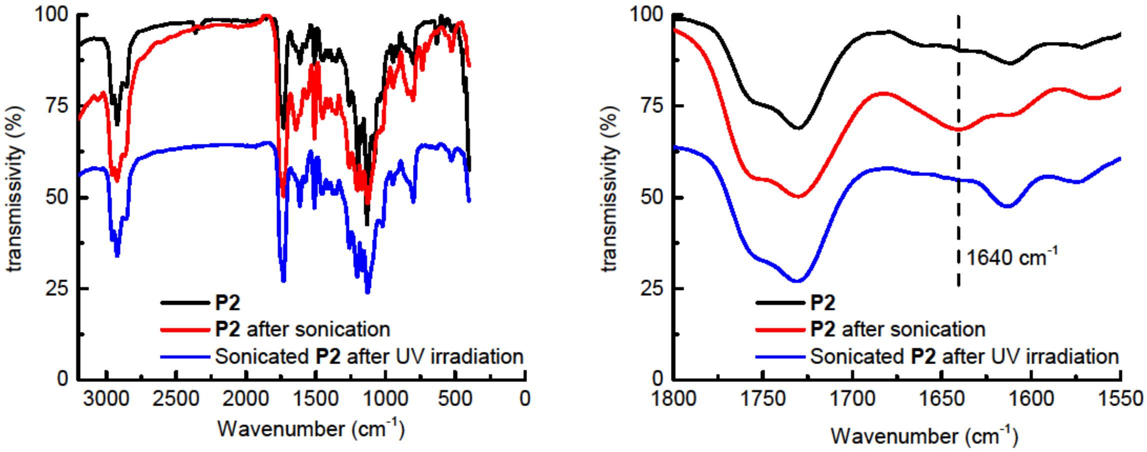


**Supplementary Figure 20**. **IR spectra of P2 and its derivatives**. The difference in the IR spectra of sonicated **P2** (red) and the material obtained by irradiating sonicated **P2** at 365 nm (blue) are indicative of dimerization of cinnamates, as shown in ref. 9.

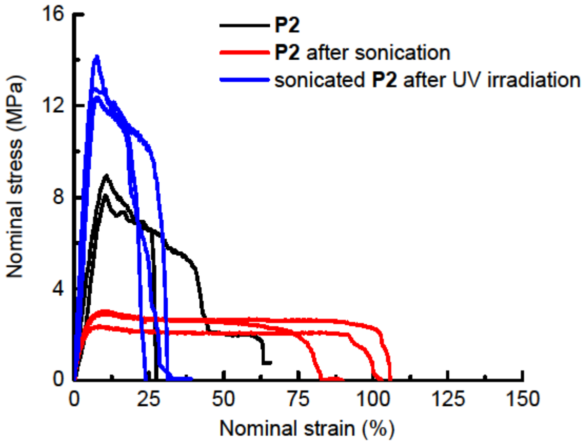


**Supplementary Figure 21. Mechanical characterization of P2 and its derivatives**. Stress/strain curves measured under macroscopic uniaxial tensile loading on films of intact **P2**; sonicated **P2** and sonicated and irradiated **P2**. Nominal stress is defined as $F/S_0$, where $F$ is the force at the grips of the tensile tester and $S_0$ is the cross sectional area before deformation; nominal strain is $\Delta L/L_0$, where $\Delta L$ and $L_0$ are the extension and the original length of the strip, respectively.

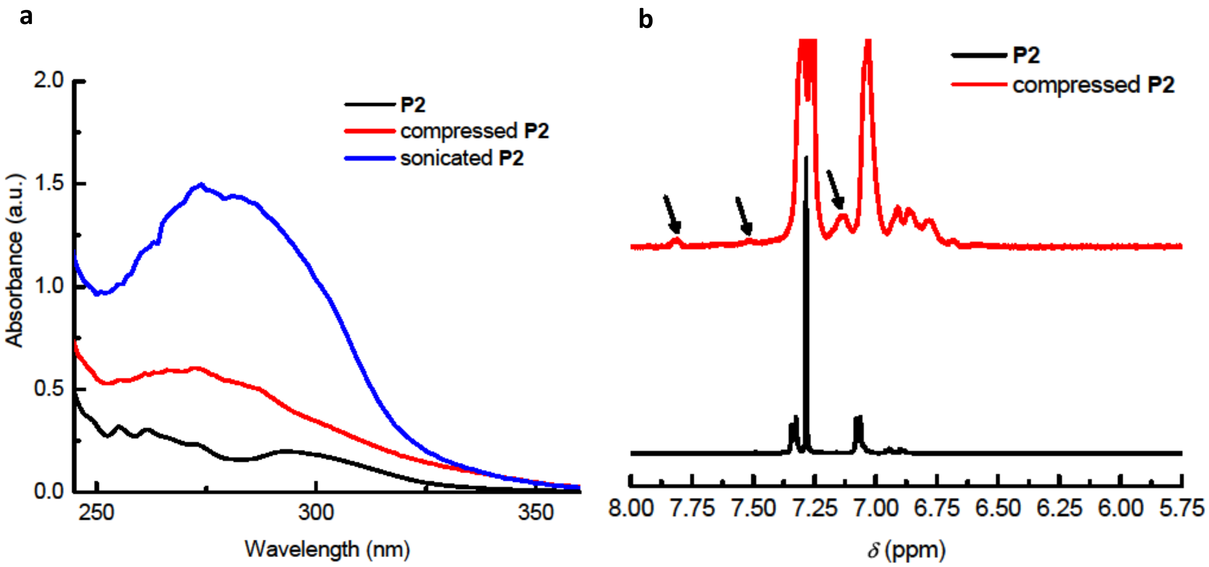


**Supplementary Figure 22**. **Evidence of dimer dissociation under compression**. (a) UV-vis spectra of THF solutions of **P2** before (black) and after uniaxial compression (5 times at 550 MPa for 5 min each, red) and after 3-h sonication (blue). The increase in the absorbance intensity at 280 nm corresponds to 5% of dimers dissociating into cinnamates under compression. (b) Solution $^1$H NMR spectra of **P2** before (black) and after uniaxial compression (5 times at 550 MPa for 5 min each). The arrows indicate the characteristic resonances of cinnamate.

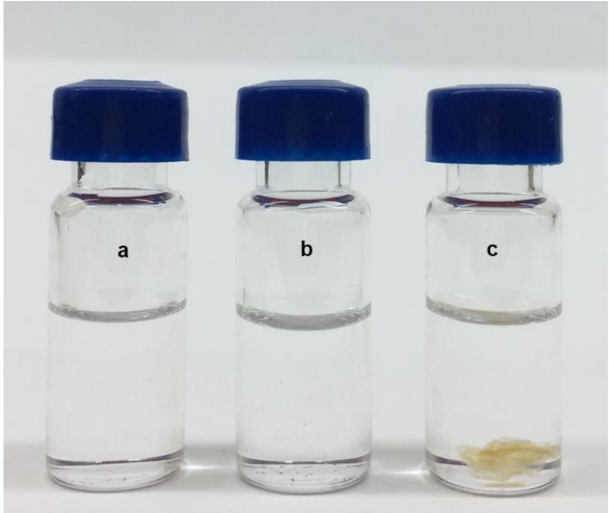


**Supplementary Figure 23**. **Optical healing yields insoluble material**. Films of **P2** before and after compressive loading are fully soluble in $CH_2Cl_2$ (a-b), but films that were loaded and then irradiated at 365 nm for 4 h are partially insoluble (compare to Supplementary Fig. 19). Irradiation of **P2** prior to mechanical loading doesn't change its solubility.

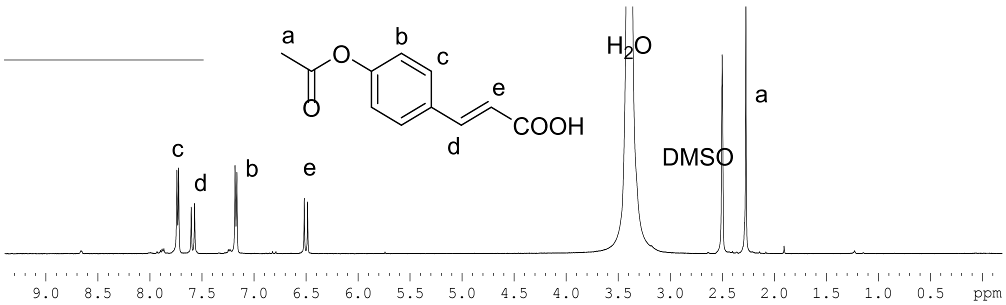


**Supplementary Figure 24. $^1$H NMR spectrum of compound 4.**

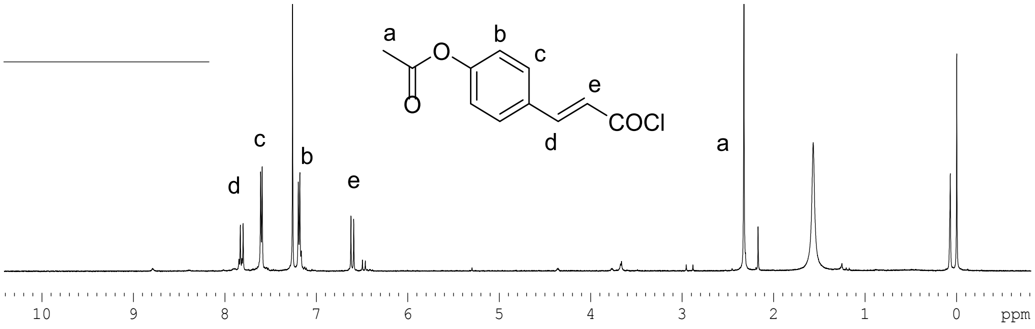


**Supplementary Figure 25. $^1$H NMR spectrum of compound 5.**

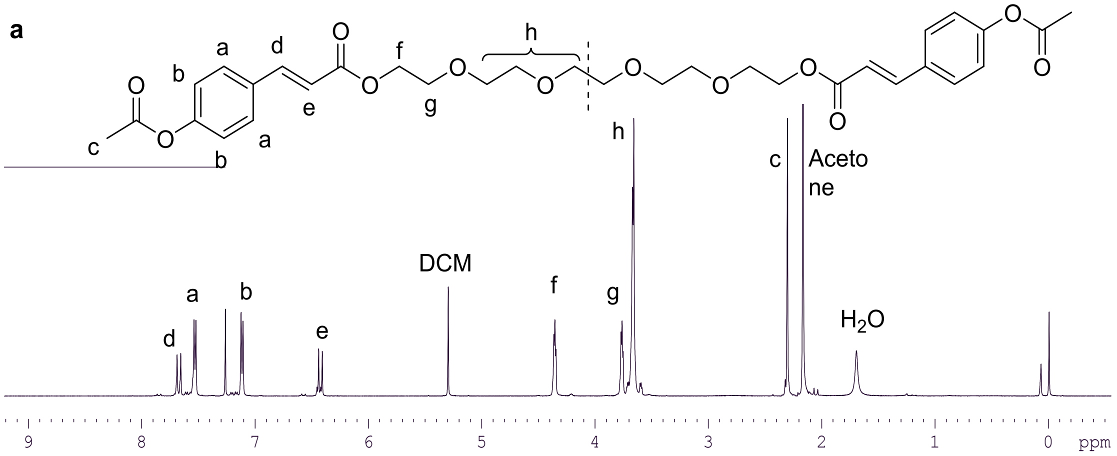


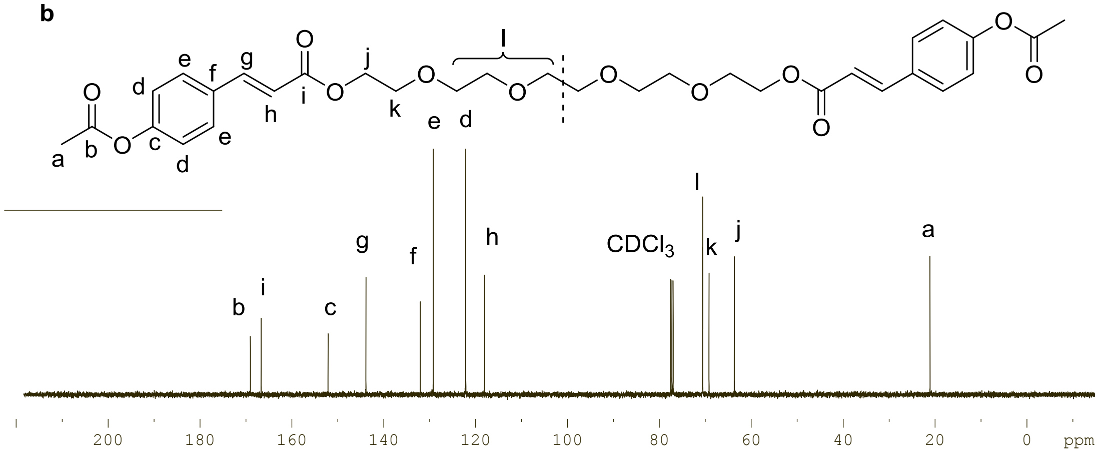


**Supplementary Figure 26. NMR spectrum of compound 6.** (a) $^{1}H$ NMR; (b) $^{13}C$ NMR.

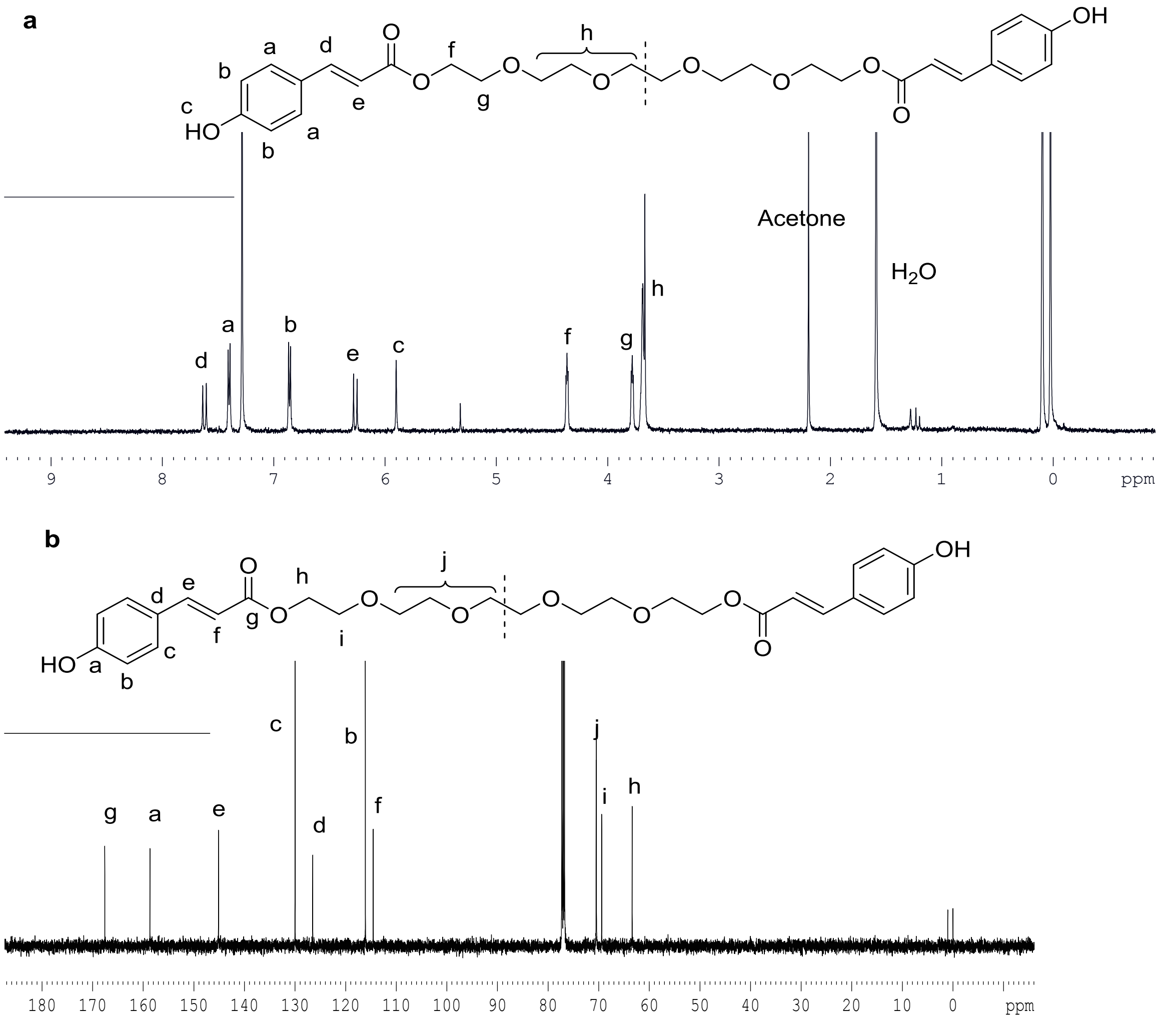


**Supplementary Figure 27. NMR spectrum of compound 7.** (a) $^{1}$H NMR; (b) $^{13}$C NMR.

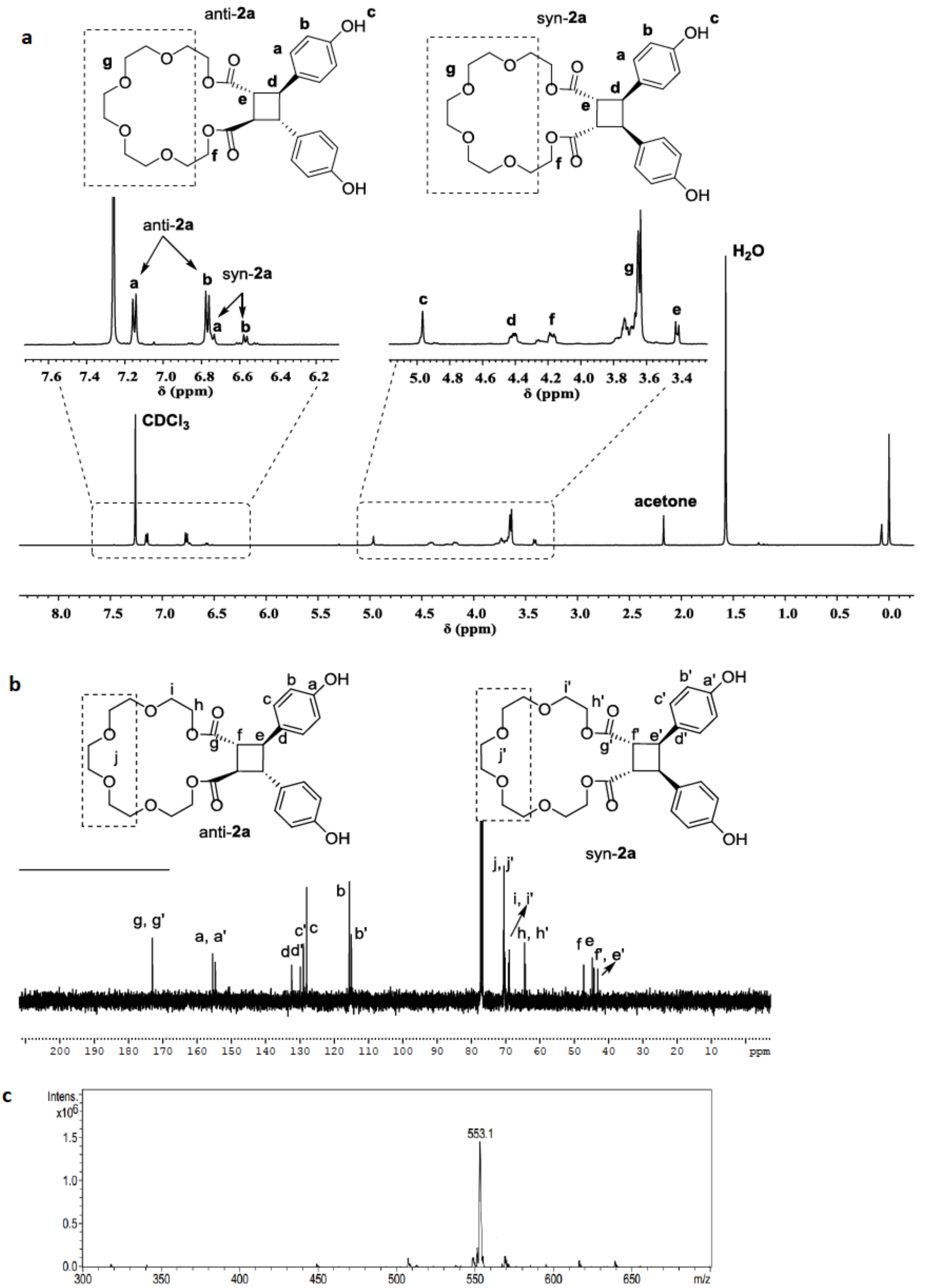


**Supplementary Figure 28. Spectroscopic characterization of 2a.** (a) $^{1}H$ NMR; (b) $^{13}C$ NMR and (c) MS.

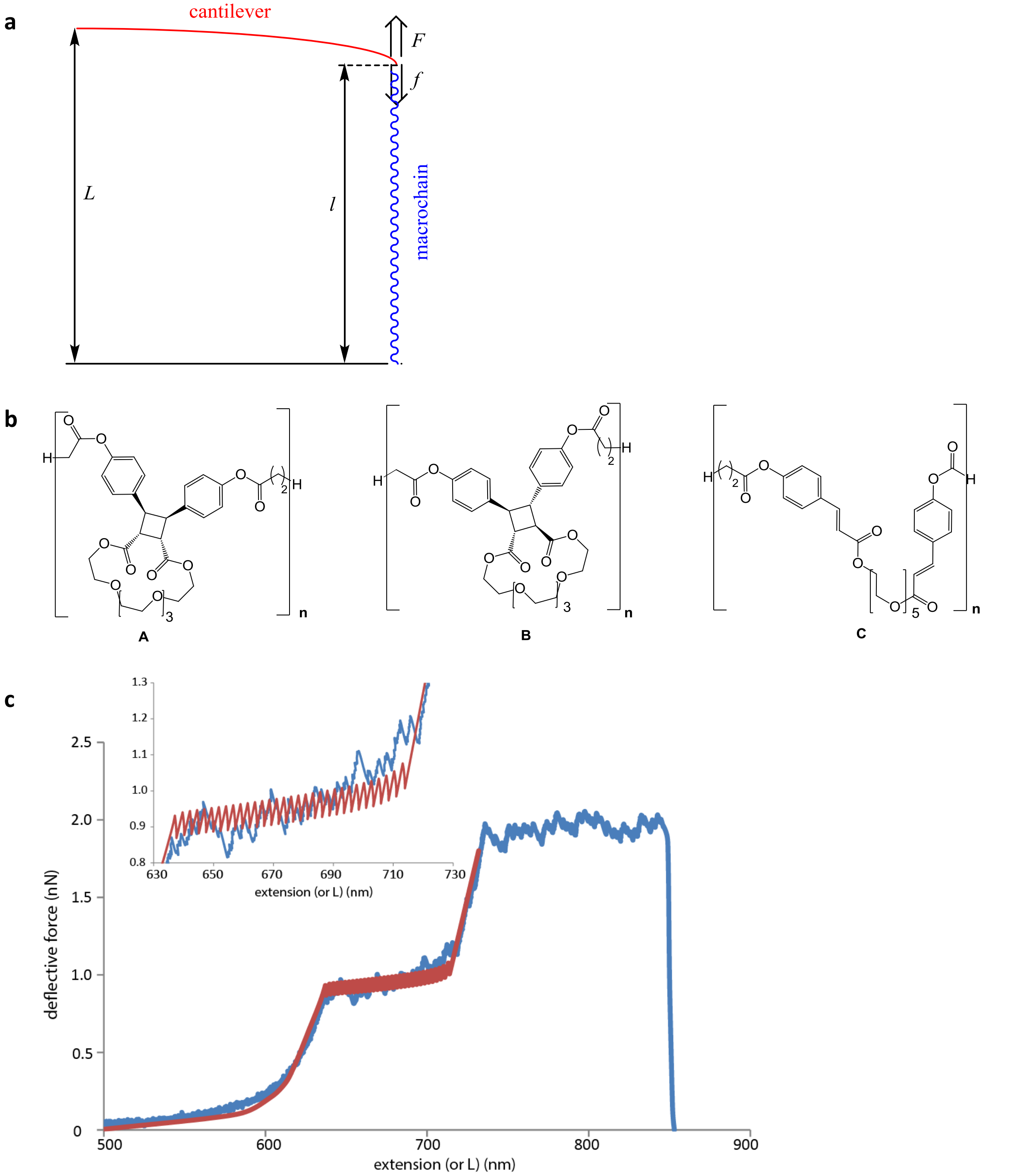


**Supplementary Figure 29. Modeling of force/extension curves**. (a) Schematic representation of the relationship between the computed parameters: *L*: measured extension; *F*: measured force, which equals the cantilever deflection force; *f*: restoring force of the macrochain (which, equals *F* on the second time scale); *l*: separation of the chain termini. (b) Chemical structures of polymers used in simulations. (c) comparison of calculated (red) and experimental force/extension curves. See the Calculation of single-chain force/extension curves of **P2** section below for further details.

**Supplementary Table 1.** Comparison of electronic energies (in kcal mol$^{-1}$) of conformers of the 1$^{st}$ (TS1) and 2$^{nd}$ (TS2) transition states of thermal dissociation of cis-cyclobutane-1,2-dicarboxylic acid at uCCSD/jun-cc-pVTZ//uCCSD/6-31G(d), uMPW1K/6-311+G(d) and uB3LYP/6-311+G(d) levels of theory. The blue arrows in the structures show the atomic motion comprising the reactive mode.

| | uMPW1K/6-311+G(d) geometry | uCCSD/jun-cc-pVTZ //uCCSD/6-31G(d) | uMPW1K/6-311+G(d) | uB3LYP/6-311+G(d) |
|---|---|---|---|---|
| TS1 | 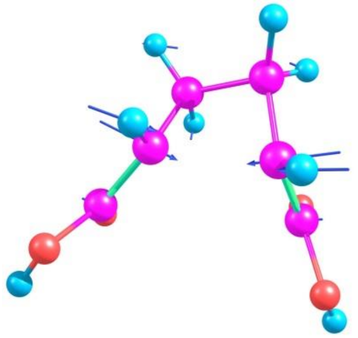 | 50.8 | 47.1 | 43.4 |
| TS2 | 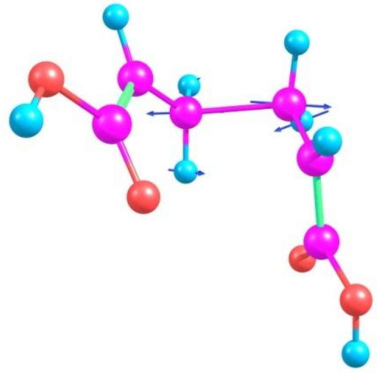 | 53.4 | 52.4 | 42.2 |
| TS2 | 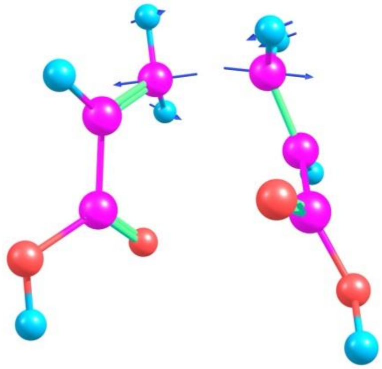 | 51.8 | 51.6 | 42.2 |
| | **Average error** | | **1.6** | **9.4** |

**Supplementary Table 2**. Summary of the mechanical properties of **P2** before and after sonication, and after sonication and optical healing under macroscopic uniaxial tensile loading.

| Trial | Modulus (MPa) | | | Ultimate strength (MPa) | | | Elongation at break (%) | | | Toughness (J m$^{-3}$) | | |
|---|---|---|---|---|---|---|---|---|---|---|---|---|
| | **P2** | Sonic. **P2** | Healed/sonic **P2** | **P2** | Sonic. **P2** | Healed/sonic **P2** | **P2** | Sonic. **P2** | Healed/sonic **P2** | **P2** | Sonic. **P2** | Healed/sonic **P2** |
| 1 | 79.5 | 59.9 | 235.6 | 8.09 | 2.34 | 14.2 | 45 | 92 | 30 | 1.51 | 1.89 | 3.09 |
| 2 | 99.1 | 60.3 | 227.4 | 8.96 | 2.90 | 12.3 | 29 | 82 | 27 | 2.80 | 1.95 | 2.29 |
| 3 | | 60.9 | 251.4 | | 2.96 | 12.7 | | 102 | 23 | | 2.64 | 2.26 |
| average | 89.5 | 60.4 | 238.1 | 8.53 | 2.73 | 13.1 | 37 | 92 | 27 | 2.16 | 2.16 | 2.54 |
| Std dev. | 13.9 | 0.5 | 12.2 | 0.62 | 0.34 | 1.0 | 11 | 10 | 4 | 0.91 | 0.42 | 0.57 |

# Supplementary Methods

## General experimental procedures.

5-Nitrosalicylaldehyde, aluminum trichloride, methacrylic acid, *N,N*-dimethylthiocarbamoyl chloride, 1,4-diazabicyclo[2.2.2]-Octane, N-Ethylmaleimide were purchased from Energy. Glutaric acid (99.999%), 2-Iodoethanol, 2,3,3-Trimethylindoleniune, dibutyltin dilaurate, 1,6-hexyldiisocyanate, dibutyltin dilaurate, PTHF ($M_n$ = 2000 g mol$^{-1}$), maleic anhydride were obtained from Alladin. Chloromethylmethyl ether was obtained from Alfa. THF was dried with Na before use. Dichloromethane, DMF, toluene were distilled over $CaH_2$ under nitrogen. All the other reagents were purchased from Sinopharm and used without further purification.

$^1$H NMR and $^{13}$C NMR spectra were recorded in either $CDCl_3$ or DMSO and referenced to the residual solvent signals on a 500 MHz Brucker Avance II spectrometer at 25 °C. All chemical shifts were given in ppm (*δ*) as singlet (d), triplet (t), quartet (q), multiplet (m), or broad (br).

Gel permeation chromatography (GPC) data were calibrated on two in series columns (7.8 × 300 mm, 2 GMHHRM17932 and 1 GMHHRH17360) with THF (HPLC grade) as eluent at 40 $^o$C with a LC-20AD pump. The facility was equipped with two detectors (RID-10A refractive index detector; SPD-20A UV detector) and the molecular weight was calibrated against polystyrene standards.

Silicon nitride AFM tips (Veeco Instruments, now Bruker Nano, Santa Barbara, CA, MLCT) were used in the SMFS experiments. Before modification, the AFM tips were treated with piranha solution ($H_2SO_4$ (98%)/$H_2O_2$ (30%) = 7:3 in volume), and thoroughly rinsed with deionized water, followed by drying in an oven at 115 $^o$C for 90 min to remove any remaining water. (***Caution: Piranha solution that may result in explosion or skin burns is a very hazardous oxidant. This solution must be handled with extreme care.***) The vapor-phase deposition method was used for the silanization of clean AFM tips by placing them in the atmosphere of the APDMMS in a dry nitrogen-purged desiccator for 1.5 h at 25℃. Immediately after being taken out, the silanized tips were rinsed three times with methanol and then placed in a 110 $^o$C oven for 10 min for activation.

## Synthesis

All chemical structures and NMR spectra are shown in Supplementary Figs. 24-28.

**4-Acetoxycoumaric Acid, 4:** (E)-3-(4-hydroxyphenyl)acrylic acid (5 g, 30.4 mmol, 1 equiv.) was slowly (in 10 min) added to a cold solution of dimethylaminopyridine (DMAP) (0.09 g, 0.78 mmol, 0.025 equiv.) and acetic anhydride (4.28 mL, 45.6 mmol, 1.52 equiv.) in pyridine (10 mL). The mixture was stirred at room temperature for 1 h and then poured over crushed ice. A white solid formed when the solution was acidified (pH = 2). The white solid was collected via vacuum filtration to provide **4** (6.7 g, 27.3

mmol) in 91% yield. $^1$H NMR (DMSO, 500 MHz): δ(ppm) = 7.74 – 7.72 (d, *J* = 8.5 Hz, 2H), 7.60 – 7.57 (d, *J* = 16.1 Hz, 1H), 7.18 – 7. 16 (d, *J* = 8.6 Hz, 2H), 6.52 – 6.48 (d, *J* = 16.1 Hz, 1H), 2.27 (s, 3H).

**(E)-4-(3-chloro-3-oxoprop-1-en-1-yl)phenyl acetate, 5:** Thionyl chloride ($SOCl_2$) (1.35 ml, 38 mmol, 2.6 equiv.) and two drops of DMF were added to a stirred solution of **4** (3 g, 14.55 mmol, 1 equiv.) in dry $CHCl_3$ (20 mL). The mixture was refluxed for 6 h and allowed to cool to room temperature. The solvent and unreacted $SOCl_2$ were removed under vacuum. The acid chloride derivative **5** was directly used in the next step. $^1$H NMR ($CDCl_3$, 500 MHz): δ(ppm) = 7.83 – 7.80 (d, *J* = 15.5 Hz, 1H), 7.61 – 7.59 (d, *J* = 8.50 Hz, 2H), 7.20 -7.18 (d, *J* = 8.6 Hz, 2H), 6.22 – 6.99 (d, *J* = 15.6 Hz, 1H), 2.33 (s, 3H).

**Ethane-1,2-diyl (2E,2'E)-bis(3-(4-acetoxyphenyl)acrylate), 6** A solution of **5** (820 mg, 3.7 mmol, 2.46 equiv.) in dry $CH_2Cl_2$ (50 mL) was added dropwise to a stirred solution of pentaethylene glycol (0.9 g, 1.5 mmol, 1 equiv.) and triethylamine (TEA) (0.3 g, 3 mmol, 2 equiv.) in dry $CH_2Cl_2$ (10 mL). The reaction mixture was stirred at 25 °C overnight. The organic layer was then washed sequentially with 1 M hydrochloric acid (HCl) (25 mL), 10% sodium bicarbonate ($NaHCO_3$) (25 mL) and saturated brine (25 mL), and dried over $MgSO_4$. The solvent was evaporated under vacuum to give **6** as a white oil in 98 % yield. $^1$H NMR ($CDCl_3$, 500 MHz): δ(ppm) = 7.69 - 7.66 (d, *J* = 16 Hz, 1H), 7.54 – 7.52 (d, *J* = 8.6 Hz, 2H), 7.13 – 7.10 (d, *J* = 8.7 Hz, 2H), 6.44 – 6.41 (d, *J* = 16 Hz, 1H), 4.37 – 4.35 (t, *J* = 4.8 Hz, 2H), 3.77 – 3.75 (t, *J* = 5 Hz, 2H), 3.68 – 3.66 (m, 6H), 2.31 (s, 3H). $^{13}$C NMR ($CDCl_3$, 170 MHz): δ(ppm) = 169.01, 166.68, 152.11, 143.84, 132.03, 129.19, 122.11, 118.04, 70.59, 70.56, 69.17, 63.67, 21.05.

**Ethane-1,2-diyl (2E,2'E)-bis(3-(4-hydroxyphenyl)acrylate), 7** To a solution of **6** (2 g, 3.2 mmol, 1 equiv.) in 20 mL $CH_3OH/CH_2Cl_2$ (1:1) mixture, $K_2CO_3$ (0.9g , 6.4 mmol, 2 equiv.) was added, and the resulting solution was stirred for 20 min. Saturated aqueous NaCl was added to the mixture. The product was extracted with $CH_2Cl_2$ and dried over anhydrous $Na_2SO_4$. The solvent was removed under vacuum, and the crude product was purified with column chromatography ($CH_2Cl_2$/Acetone = 5 ： 2) to give **7** (0.86 g) as a white solid in 49% yield. $^1$H NMR ($CDCl_3$, 500 MHz): δ(ppm) = 7.61 – 7.58 (d, *J* = 16 Hz, 1H), 7.38 – 7.36 (d, *J* = 8.3 Hz, 2H), 6.84 – 6.82 (d, *J* = 8.7 Hz, 2H), 6.25 – 6.22 (d, *J* = 15.8 Hz, 1H), 5.88 (s, 1H), 4.35 – 4.33 (t, *J* = 4.59 Hz, 2H), 3.76 – 3.75 (t, *J* = 4.57 Hz, 2H), 3.66 – 3.64 (m, 6H). $^{13}$C NMR ($CDCl_3$, 500 MHz): δ(ppm) =167.58, 158.66, 145.12, 129.99, 126.49, 116.09, 114.55, 70.50, 70.45, 70.43, 69.39.

## Quantum-chemical calculations

All calculations were performed with the Gaussian09.E01[10] software package. The Berny algorithm was used to locate stationary points. Very tight convergence criteria and ultrafine integration grids were used in all optimizations. Thermodynamic corrections to electronic energies of individual conformers were calculated statistical-mechanically in the harmonic oscillator/rigid rotor/ideal gas approximations, as 3RT +ZPE +$U_{vib}$ –TS, where ZPE is the zero-point energy, $U_{vib}$ is the vibrational component of the internal energy and *S* is the total entropy. Vibrational frequencies below 500 cm$^{-1}$ were replaced with 500 cm$^{-1}$ as previously recommended[11], to avoid the artifactually large contribution of such low-frequency modes to vibrational entropy. The calculations of analytical frequencies on converged constrained molecules is valid because the molecule plus its infinitely-compliant constraining potential is a stationary point.[12, 13] The free energies of ensembles were calculated as $G_{min} - RT\ln\sum g_i e^{-\Delta G_i/RT}$, where $G_{min}$ is the free energy of the conformational minimum, $\Delta G_i$ is the excess free energy of conformer *i* relative to this minimum, and $g_i$ is its degeneracy. The energy barriers separating individual strain-free conformers were <4 kcal/mol, justifying the use of Boltzmann statistics in calculating properties of ground and transition states and energies of activation. Ensemble averaging was done as $\langle\alpha\rangle = \frac{\sum \alpha_i g_i e^{-G_i\beta}}{\sum g_i e^{-G_i\beta}}$, where α is the quantity of interest (e.g., end-to-end distance) and the remaining terms are defined above.

The converged wavefunctions were stable as determined by outcome of the testing with the "stable" key word in Gaussian. All converged conformers of the reactant or intermediate states had 0 imaginary frequencies and all converged conformers of the transition states had a single imaginary frequency with the nuclear motion consistent with dissociation/rotation as appropriate. Unconstrained conformational ensembles of **1**-**3** were built systematically as previously described.[14, 15] All unique conformers of every stationary state of **1** were fully optimized at the uMPW1K/6-31+G(d) level. For macrocycles **2**-**3**, conformers of the reactant, TS1, TS2, TS1', TS2', Int and Int' (with one or both scissile bonds constrained for all species other than the reactant) were first generated at the MM3 level, followed by constrained optimization at uBLYP/6-31+G(d), and constrained reoptimization of the unique BLYP conformers within 1.5 kcal $mol^{-1}$ from each conformational minima at the uMPW1K/6-31+G(d) level. Of these, all conformers of the transition states and intermediates within 1 kcal $mol^{-1}$ from the conformational minima were fully reoptimized (analytical frequencies were calculated before and after optimization of all transition state conformers and some intermediates, where initial optimization converged to a reactant because of a poor initial estimate of the Hessian). Force-dependent properties of individual conformers and the conformational ensembles were calculated following the described procedures. [14, 15]

## Calculation of single-chain force/extension curves of P2

No analytical solution describes the force/extension curve or the evolution of the composition of the chain during a dynamic single-molecule force experiment.[14, 16] Consequently, force/extension curves were calculated by incrementing the value of the stretching time, *t*, and control parameter, *L*, (figure on the right) and calculating all other parameters determining the behavior of the chain (chain length, *l*, restoring force of the chain, *f*, chain composition, *x*; bending force of the cantilever, *F* and the survival probability, *s*) for each sequential value of *t* and *L* as described below. Force/extension curves for dissociation of syn dimers were calculated independently from those for anti dimers (in the former case, the simulation stopped when the final syn dimer dissociated; in the latter case, the simulations started with copolymer of *EE* diene and anti dimer). Because isomerization of each monomer is a stochastic process, for a chain containing *n* dimers we calculated $\sim 10^5 n^{3/2}$ (or $10^8$, whichever was smaller) individual force/extension curves. In each simulation a unique combination of *n* monotonically decreasing random numbers from 0.9999 to $10^{-4}$ was used to define at which simulation step each dimer dissociated (below we call this set of *n* numbers probability vector, S). These sets of random numbers were generated and converted to the total relative probability that each calculated force/extension curve would appear among all generated curves as described below.

The force-extension curve of a chain comprised of *a* syn dimers, *b* anti dimers and *c E,E* dienes was calculated as $l(f)=a(l(\mathbf{A}_{n=2})+l(\mathbf{A}_{n=4})/2+l(\mathbf{A}_{n=6})/3)/6+ b(l(\mathbf{B}_{n=2})+l(\mathbf{B}_{n=4})/2+l(\mathbf{B}_{n=6})/3)/6 + c(l(\mathbf{C}_{n=1})+l(\mathbf{C}_{n=2})/2)/2$, where $l(\mathbf{X}_{n=y})$ is the ensemble average separation of the C atoms of the terminal Me groups (i.e., $_{Me}C^{\cdots}C_{Me}$) as a function of the externally applied force for one of the compounds shown above, which are repeat units of copolymer **P2** before or after dimer dissociation. In other words, our calculations of the measured force/extension curves used scaled $_{Me}C^{\cdots}C_{Me}$ distances extrapolated from ensemble-averaged $_{Me}C^{\cdots}C_{Me}$ distances of repeat units of polymer **P2** before and after mechanochemical reactions calculated at the BLYP/6-31+G* level in vacuum. Some of the force/extension curves for the extreme cases (e.g., *c*=0 or *a*=0) are illustrated in Supplementary Fig. 11 as dashed red, blue and green curves).

At the $i^{th}$ step of the calculation $L_i = L_{i-1} + v\Delta t_i$ ($v$ = 1 μm/s is the experimental stretching rate and $\Delta t_i$ is the time increment of the $i^{th}$ step) and the composition of the chain was $x_i$ (which defines the number of syn, anti and *E,E* monomers, i.e., $x_i=[a_i,b_i,c_i]$ using the notations above). If the condition of mechanical equilibrium was satisfied ($|f_i| = |F_i|$, see below), the end-to-end separation of the chain was found numerically be solving the equation $l_i = L_i - f(l_i,x_i)/\lambda$, where $f(l,x)$ is the force-extension curve for a chain

of composition $x$ derived from quantum-chemical calculations as described above and $\lambda$ is the harmonic compliance of the cantilever. From $l_i$, $f_i$ (the restoring force of the chain) was found using the $f(l,x)$ correlation. From $f_i$, the expectation value of the survival probability of the dimer by time $t_i$ from the start of the experiment, $s_i$, is found as $s_i = s_{i-1}(1-k(f_i)\Delta t_i)$, where $k(f_i) = \frac{k_B T}{h} e^{-\Delta G_t^{\ddagger}(f_i)/RT}$ is the rate constant of dissociation at the restoring force $f_i$ and $\Delta G^{\ddagger}_t(f_i)$ are plotted as green curves on the right panel of Supplementary Fig. 5. If $s_i$ exceeded a predefined value, the simulation progressed to the (i+1)$^{th}$ step. Otherwise, the composition of the chain was changed by reducing the number of the appropriate isomer of the dimer by one and increasing the number of the *E,E* dienes by 1. Because a large object such as an AFM tip moves much slower than the rate at which the chain reaches internal mechanical equilibrium after one of its monomers reacted, for a few steps immediately after dimer dissociation the absolute value of the restoring force of the chain, $|f_i|$ is less than the force corresponding to the instantaneous deflection of the cantilever, $|F_i|$ which is evidenced in the experiments by short segments of force/extension curves where the extension increases while the force decreases. Consequently, for a few steps following the dissociation the force of the cantilever was calculated as $|F_i|=|F_{i-1}|-\alpha\Delta t_i(|F_{i-1}|-|f(l_i,x_i)|)$, where $\alpha$ is a coefficient whose value was adjusted to reproduce the rate at which mechanical equilibrium between the cantilever and the stretched chain re-establishes. This formula was applied as long $|f(L_i,x_i)|<|F_{i-1}|+10$ pN, after which the mechanical equilibrium was assumed to have re-established. A comparison of an experimental force extension curve (blue) and a calculated $L(F)$ curve (red) for an experimentally studied chain comprised of 32 syn dimers and 308 anti dimers is shown below (in this simulation, only syn dimers were reacted).

In each simulation the step at which a dimer was dissociated was determined by comparing the expectation value of the survival probability with the corresponding number from the probability vector. For each chain containing $n$ syn dimers or $n$ anti dimers we generated ~$10^5 n^{3/2}$ unique vectors (i.e., sets of $n$ monotonically decreasing random numbers selected between 0.9999 and $10^{-4}$). The example above corresponds to the highest-probability $L(F)$ curve and was generated using the following set of random numbers to define the survival probabilities at which each subsequent dimer dissociated (for space, only 3 significant digits are listed; 16 significant digits were used in simulations): 0.998; 0.996; 0.994; 0.991; 0.989; 0.986; 0.983; 0.980; 0.977; 0.973; 0.969; 0.965; 0.960; 0.954; 0.949; 0.942; 0.935; 0.926; 0.917; 0.906; 0.894; 0.879; 0.862; 0.840; 0.814; 0.782; 0.739; 0.681; 0.600; 0.476; 0.275; $10^{-4}$. The relative weight of the j$^{th}$ force/extension curves in the collection of the $10^5 n^{3/2}$ simulated curves was calculated as

$$\left(1-s_{j,1}^{n}\right)s_{j,n}\prod_{i=2}^{n}\left(s_{j,i}^{n-i+1}-s_{j,i-1}^{n-i}\right) \Bigg/ \sum_{j=1}^{10^5 n^{1.5}}\left(\left(1-s_{j,1}^{n}\right)s_{j,n}\prod_{i=2}^{n}\left(s_{j,i}^{n-i+1}-s_{j,i-1}^{n-i}\right)\right) \qquad (1)$$

where $s_{j,i}$ is the i$^{th}$ number from the vector above. Physically, this relative weight corresponds to the probability of the j$^{th}$ curve to be observed if the experiment were repeated under identical conditions $10^5 n^{3/2}$ times. The curve illustrated above had the calculated relative probability of $3.13\times10^{-7}$.

For chains with fewer than 15 reactive dimers, the set of probability vectors were generated using the rand function of Matlab. After calculating their absolute weights (the numerator of the equation above), the generated vectors were sorted and only those vectors with the largest absolute weights that together accounted for 95% of the sum in the denominator of the above equation were retained. As the $n$ increases, the fraction of randomly generated probability vectors accounting for 95% of the denominator decreases very rapidly making this approach to generating probability vectors impractical for simulating force/extension curves with 15 or more equivalent reactive sites. Consequently, for chains

with more than 14 reactive dimers we used a different strategy in which we first found the probability vector corresponding to the largest possible value of the numerator by funding the roots of the system of n-1 polynomials generated by differentiating the numerator with respect to each $s_i$. We then used these values to limit the range over which each corresponding survival probability value could vary across the whole set of probability vectors. For example, the set of survival probabilities that determine when the first isomerization would occur was generated by applying the rand function to the range [0.9999:($s_{m,1}$-$s_{m,2}$)/2], where *m* signifies the highest-probability vector described above and 1 and 2 corresponds to the 1st and 2nd element of this vector. We established that the two methods produce equivalent distributions of dissociation forces by generated two sets of probability vectors (one by each method) for a chain with 10 syn (and 53 anti) and 15 syn (and 40 anti) dimers.

In calculating the distribution of single-chain forces for dissociation of anti dimers for comparison with the experiment, only the dissociation of the first *m* dimers was considered, where *m* is the number of experimentally observed dissociations.

## Supplementary references


1. Laye, P. G. Differential thermal analysis and differential scanning calorimetry. in *Principles of Thermal Analysis and Calorimetry* (ed Peter J Haines) Ch. 3, 55-93 (RSC, 2002).
2. Pomerantzev, A. L. & Rodionova, O. E. in *Chemical and Biological Kinetics* Vol.1 (ed E. B. Burlakova) 80-107 (VSP, 2005).
3. Menczel, J. D. *et al*. in *Thermal Analysis of Polymers: Fundamentals and Applications* (eds Joseph D. Menczel & R. Bruce Prime) Ch. 2, 7-239 (Wiley, 2008).
4. Lexa, D. & Leibowitz, L. in *Characterization of Materials* Vol. 1 (ed Elton N. Kaufmann) 362-373 (Wiley, 2003).
5. Westerhout, R. W. J., Waanders, J., Kuipers, J. A. M. & van Swaaij, W. P. M. Kinetics of the low-temperature pyrolysis of polyethene, polypropene, and polystyrene. Modeling, experimental determination, and comparison with literature models and data. *Ind. Eng. Chem. Res.* **36**, 1955-1964, doi:10.1021/IE960501M (1997).
6. Prime, R. B., Bair, H. E., Vyazovkin, S., Gallagher, P. K. & Riga, A. in *Thermal Analysis of Polymers: Fundamentals and Applications* (eds Joseph D. Menczel & R. Bruce Prime) Ch. 2, 241-317 (Wiley, 2008).
7. Reich, L. & Levi, D. W. Dynamic thermogravimetric analysis in polymer degradation. *Macromol. Rev.* **1**, 173-275, doi:10.1002/pol.1967.230010106 (1967).
8. Jurd, L. The detection of aromatic acids in plant extracts by the ultraviolet absorption spectra of their ions. *Arch. Biochem. Biophys.* **66**, 284-288, doi:10.1016/S0003-9861(57)80003-4 (1957).
9. Cho, S.-Y., Kim, J.-G. & Chung, C.-M. A fluorescent crack sensor based on cyclobutane-containing crosslinked polymers of tricinnamates. *Sens. Actuator B-Chem.* **134**, 822-825, doi: 10.1016/j.snb.2008.06.042 (2008).
10. Gaussian 09 Revision E.01, M. J. Frisch, G. W. Trucks, H. B. Schlegel, G. E. Scuseria, M. A. Robb, J. R. Cheeseman, G. Scalmani, V. Barone, G. A. Petersson, H. Nakatsuji, X. Li, M. Caricato, A. Marenich, J. Bloino, B. G. Janesko, R. Gomperts, B. Mennucci, H. P. Hratchian, J. V. Ortiz, A. F. Izmaylov, J. L. Sonnenberg, D. Williams-Young, F. Ding, F. Lipparini, F. Egidi, J. Goings, B. Peng, A. Petrone, T. Henderson, D. Ranasinghe, V. G. Zakrzewski, J. Gao, N. Rega, G. Zheng, W. Liang, M. Hada, M. Ehara, K. Toyota, R. Fukuda, J. Hasegawa, M. Ishida, T. Nakajima, Y. Honda, O. Kitao, H. Nakai, T. Vreven, K. Throssell, J. A. Montgomery, Jr., J. E. Peralta, F. Ogliaro, M. Bearpark, J. J. Heyd, E. Brothers, K. N. Kudin, V. N. Staroverov, T. Keith, R. Kobayashi, J. Normand, K. Raghavachari, A. Rendell, J. C. Burant, S. S. Iyengar, J. Tomasi, M. Cossi, J. M. Millam, M. Klene, C.

Adamo, R. Cammi, J. W. Ochterski, R. L. Martin, K. Morokuma, O. Farkas, J. B. Foresman, and D. J. Fox, Gaussian, Inc., Wallingford CT, 2016.

11. Davis, D. A., *et al.* Force-induced activation of covalent bonds in mechanoresponsive polymeric materials. *Nature* **459**, 68-72, doi:10.1038/nature07970 (2009).
12. Kucharski, T. J. & Boulatov, R. The physical chemistry of mechanoresponsive polymers. *J. Mater. Chem.* **21**, 8237-8255, doi:10.1039/c0jm04079g (2011).
13. Kochhar, G. S., Heverly-Coulson, G. S. & Mosey, N. J. in *Polymer Mechanochemistry* (ed Roman Boulatov) 37-96 (Springer International Publishing, 2015).
14. Akbulatov, S., Tian, Y. & Boulatov, R. Force-reactivity property of a single monomer is sufficient to predict the micromechanical behavior of its polymer. *J. Am. Chem. Soc.* **134**, 7620-7623, doi:10.1021/ja301928d (2012).
15. Tian, Y. & Boulatov, R. Quantum-chemical validation of the local assumption of chemomechanics for a unimolecular reaction. *ChemPhysChem* **13**, 2277-2281, doi:10.1002/cphc.201200207p (2012)
16. Huang, Z. & Boulatov, R. Chemomechanics: chemical kinetics for multiscale Phenomena. *Chem. Soc. Rev*. **40**, 2359-2384, doi:10.1039/c0cs00148a (2011).
